# Damage to the Ventromedial Prefrontal Cortex Impairs Learning from Observed Outcomes

**DOI:** 10.1093/cercor/bhv080

**Published:** 2015-04-24

**Authors:** Dharshan Kumaran, David E. Warren, Daniel Tranel

**Affiliations:** 1 Institute of Cognitive Neuroscience, University College London, London, UK; 2 Department of Neurology, Carver College of Medicine; 3 Department of Psychology, University of Iowa, Iowa City, IA, USA

**Keywords:** decision-making, learning, observational, reversal, ventromedial prefrontal cortex

## Abstract

Individuals learn both from the outcomes of their own internally generated actions (“experiential learning”) and from the observation of the consequences of externally generated actions (“observational learning”). While neuroscience research has focused principally on the neural mechanisms by which brain structures such as the ventromedial prefrontal cortex (vmPFC) support experiential learning, relatively less is known regarding how learning proceeds through passive observation. We explored the necessity of the vmPFC for observational learning by testing a group of patients with damage to the vmPFC as well as demographically matched normal comparison and brain-damaged comparison groups—and a single patient with bilateral dorsal prefrontal damage—using several value-learning tasks that required learning from direct experience, observational learning, or both. We found a specific impairment in observational learning in patients with vmPFC damage manifest in the reduced influence of previously observed rewards on current choices, despite a relatively intact capacity for experiential learning. The current study provides evidence that the vmPFC plays a critical role in observational learning, suggests that there are dissociable neural circuits for experiential and observational learning, and offers an important new extension of how the vmPFC contributes to learning and memory.

## Introduction

To make optimal decisions, individuals must learn the value of stimuli in an ever-changing environment and use this information to guide choice behavior. While much research has been conducted regarding the role of the ventromedial prefrontal cortex (vmPFC) in value-guided choices based on learning from the consequences of one's own actions (Damasio et al. 1990; Damasio 1994; Bechara et al. 2000; Rolls 2004; Rangel et al. 2008; Kable and Glimcher 2009; Rushworth et al. 2011; Rudebeck and Murray 2011a), less is understood about the neural mechanisms by which individuals learn the value of stimuli in the environment through the passive observation of externally generated actions and their consequences. Interestingly, however, a recent neuroimaging investigation (Burke et al. 2010) found that observational learning in a social context was related to brain activation in specific portions of prefrontal cortex, specifically the vmPFC and dorsolateral prefrontal cortex (dlPFC). Notably, activation of the 2 regions was related to dissociable aspects of observational learning: vmPFC activation was linked specifically to prediction errors at the time of “outcome” (i.e., outcome prediction error: Differences between expected and observed reward), whereas activity in dlPFC reflected prediction errors at the time of “choice” (i.e., action prediction error: Differences between expected and observed action).

In the current study, we tested the hypothesis that the vmPFC is necessary for an intact capacity for observational learning, by examining the performance of patients with focal lesions of the vmPFC. While the notion of observational learning is multifaceted (Hopper 2010; Zentall 2012), here we focus on a specific component of this process: The ability of individuals to learn from passively watching the consequences of actions that were externally generated (i.e., by a computer player whose choices were random, and known to be so by participants). Our experimental paradigm, therefore, differs from previous studies in the field of social decision-making that have examined how individuals learn from and imitate the behavior of intelligent social agents (Sanfey 2007; Hampton et al. 2008; Behrens et al. 2009; Burke et al. 2010; Seo and Lee 2012; Boorman et al. 2013; Chang et al. 2013). Instead, our procedure has closer parallels with experimental paradigms used to investigate the differential contributions of the striatum and hippocampus to feedback-based and observational learning, respectively, in the memory literature (Poldrack et al. 2001; Shohamy et al. 2004)—and also the “ghost” conditions used previously to examine the nonsocial components of observational learning—for example, where the observer passively watches a remotely controlled door being moved randomly either to the right or to the left by an experimenter to reveal a food reward (Hopper et al. 2008; Hopper 2010).

**Figure 1.**
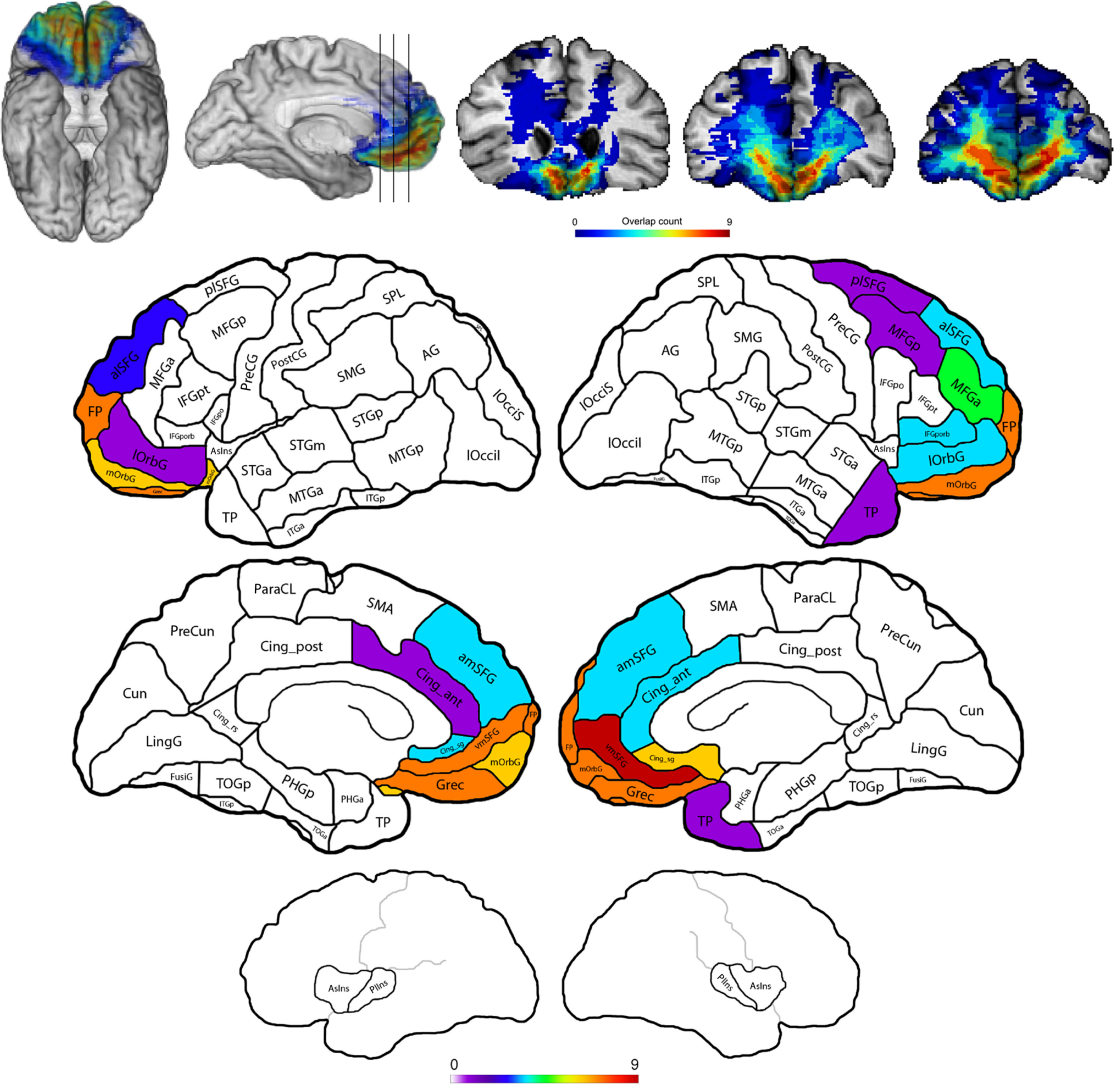
Lesion overlap maps for the vmPFC group. Top: voxel-wise overlap maps of lesion overlap mapped into a template space. “Hotter” colors indicate a greater overlap of lesions across participants (distinct color scales are used in the top and bottom panels). From left: a three-dimensional reconstruction of the lesion overlaps viewed from the bottom and right medial perspectives, with black vertical lines indicating the coronal slice locations: caudal coronal slice; middle coronal slice; and rostral coronal slice. On the far left, an “axial” slice (see Supplementary Fig. 1 for further axial slices). Bottom: a schematic region-of-interest (ROI)-based lesion overlap map illustrating gray matter lesion overlap counts in ROIs for all patients whose lesion encompassed at least 10% of ROI gray matter. See Supplementary Figure 1 for BDC lesion overlap map, and Supplementary Tables 1–4 for patient demographics and anatomical information.

**Figure 2.**
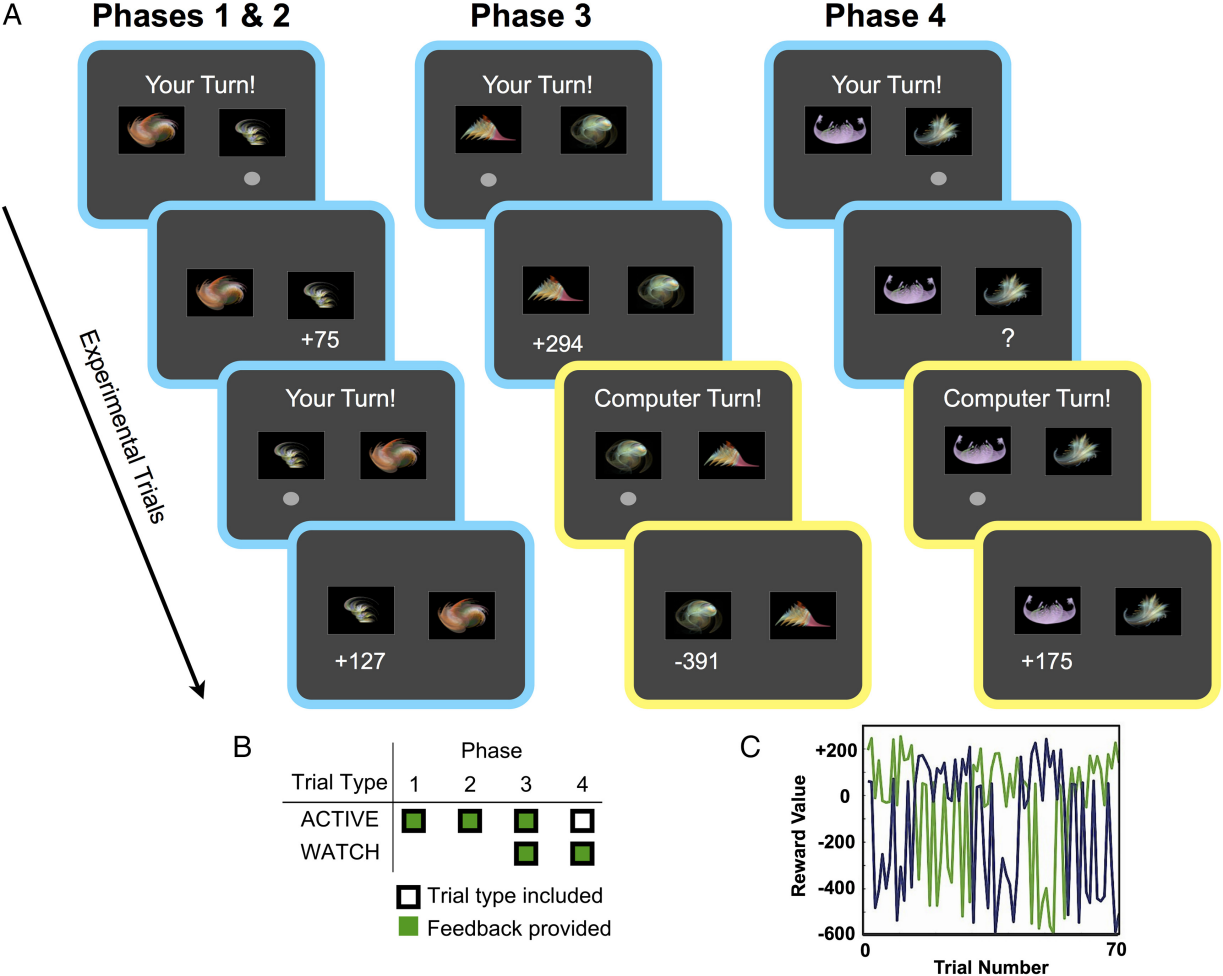
Overview of experimental phases in the probabilistic task. (*A*) During “ACTIVE” trials (blue border, for illustration), participants chose a fractal and then either received feedback concerning the outcome of their choice (phases 1–3), or received no feedback (phase 4). During “WATCH” trials (yellow border, for illustration), participants observed a computer player making a choice, followed by the outcome of this choice. Participants could learn by observing the outcomes during WATCH trials—though the actual choices of the computer were known to be uninformative (i.e., random). The composition of individual phases differed as to whether WATCH trials were included (phases 3–4), and whether the participant received feedback as to the outcomes of their own choices during ACTIVE trials (this was present in phases 1–3; no experiential outcome feedback was presented in phase 4 although participants could still observe computer outcomes during WATCH trials). Reversals occurred in phases 2–4, but not in phase 1. Different pairs of fractals were used in each phase. See Materials and Methods. (*B*) Schematic illustration of trial type included (ACTIVE/WATCH) and feedback (yes or no) in each of the 4 experimental phases. (*C*) A typical reward schedule for one phase and one vmPFC patient, illustrating how the reward values of the 2 fractals (blue and green lines) fluctuated over trials. Note that 4 reversals were triggered in this example, through the participant choosing the good stimulus in the last 9 of 10 preceding choices.

We assessed the performance of a cohort of patients who had focal damage to the vmPFC (vmPFC group), as well as healthy normal comparison (NC), brain-damaged comparison (BDC) groups, and a single case with damage to the bilateral dorsal prefrontal cortex (dPFC; Fig. 1 and see Supplementary Figs 1–3 and Tables 1–4) in a series of relatively simple two-arm bandit tasks (Fig. 2) involving either “experiential learning” alone (phases 1 and 2), “observational learning” alone (phase 4), or both types of learning interleaved (phase 3). Although previous neuropsychological studies have assessed whether vmPFC patients perform decision-making tasks differently from comparison participants by using coarse aggregate measures of behavior (Rolls et al. 1994; Fellows and Farah 2003; Hornak et al. 2004; Fellows and Farah 2007; Tsuchida et al. 2010), here we applied fine-grained analytic procedures to characterize choice behavior (Lau and Glimcher 2005; Kennerley et al. 2006; Rutledge et al. 2009; Kovach et al. 2012). Critically, this analysis allowed us to distinguish the unique influences of past actions and outcomes (both “experienced” and “observed”) on subsequent behavior. Based on recent neuroimaging findings relating to observational learning in the social domain (Burke et al. 2010), we predicted that patients with vmPFC lesions would show an impairment in observational learning in the context of our experimental paradigm, reflected in the reduced influence of observed outcomes on participants' behavior. Indeed, our study was specifically configured to test the prediction that the vmPFC makes a critical contribution to learning from observed outcomes, because outcomes were informative whereas observed choices were not (i.e., the choices of the computer player were random).

## Materials and Methods

### Participants: Neuropsychology

**Figure 3.**
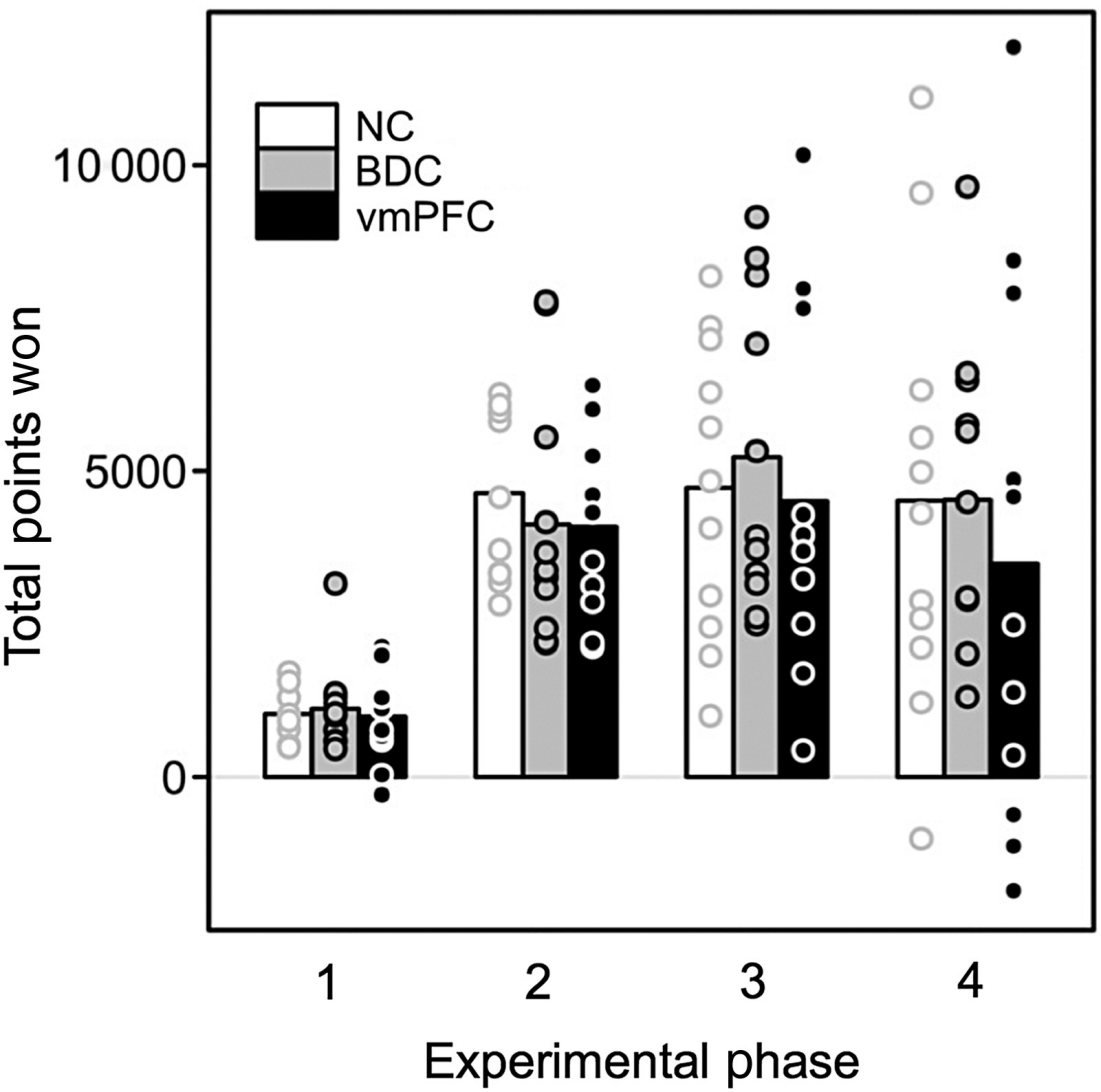
Overall performance across the 4 phases of the probabilistic task. The measure of total points is calculated as the cumulative gain above that of a random player (i.e., earning the average value of the 2 fractals on each trials—see Materials and Methods). Performance of vmPFC patients (black), BDC patients (gray), and comparison participants (white). Note that phase 1 was shorter in terms of the number of trials. Circles indicate the performance (i.e., number of points won) by individual participants. See Supplementary Table 5 for full details of performance indices.

The target patient group for this study (*N* = 11) consisted of adults with damage to the vmPFC who have generally intact psychometric intelligence, memory, and executive function (see Supplementary Table 1). A group of neurologically and psychiatrically normal adults (*N* = 11) were enrolled as healthy comparison (NC) participants, and a group of psychiatrically normal adults with focal brain damage (*N* = 11) served as BDC participants. All 3 groups were well matched for average age (mean [SD] years of age: NC = 64.25 [6.47]; BDC = 60.36 [12.39]; and vmPFC = 64.09 [7.99]) and education (years of education: NC = 15.33 [2.50]; BDC = 16.45 [2.34]; and vmPFC = 14.73 [2.19]), and equal numbers of men and women were enrolled in each group (6 female and 5 male; see Supplementary Tables 1 and 2). We also tested 1 patient with damage to the dPFC (see Supplementary Table 2 and Fig. 3) that included bilateral dorsolateral and dorsomedial PFC. A single case was studied because of the rarity of bilateral lesions of this region, in contrast to unilateral lesions. The single dPFC patient closely matched the other groups demographically and neuropsychologically, except for a specific impairment in executive function (i.e., Wisconsin card sorting task; see Supplementary Table 2).

Patients (vmPFC, BDC, and dPFC) were selected from the Patient Registry of the Division of Cognitive Neuroscience at the University of Iowa. All patients conformed to the inclusion criteria of the Patient Registry. Specifically, they had focal, stable lesions that could be clearly identified on magnetic resonance (MR) or computerized tomography (CT) scans, and they were free of dementia, psychiatric disorder, and substance abuse. All participants were free of significant intellectual impairments. The patients had no premorbid histories of abnormal social conduct, emotional maladjustment, or other psychological disturbance. Neuropsychological, neuroanatomical, and experimental studies were all conducted in the chronic phase of recovery, >3 months after lesion onset. All lesions were acquired in adulthood, and were stable since the patient's most recent neuroimaging session and corresponding lesion analysis (see below). The neuropsychological tests reported in Supplementary Tables 1 and 2 were administered after participating patients had enrolled in the Patient Registry, but prior to their participation in this project. At recruitment, the neuropsychological profiles of all patients were stable since their most recent examinations, as supported by informal observation during enrollment and test sessions. NC participants were recruited from the surrounding community through advertisement, and they were compensated for their participation. The study was approved by the Human Subjects Committee of the University of Iowa, and all participants gave informed consent before completing the study in compliance with the Declaration of Helsinki. Data were collected at the University of Iowa Hospitals and Clinics, and were de-identified before being transmitted to other authors for collaborative consideration and analysis.

### Participants: Lesion Description and Analysis

#### vmPFC Group

Neuroanatomical analysis was based on MR data for 5 vmPFC participants and CT data for 5 vmPFC participants (i.e., those with surgically implanted clips). All structural neuroimaging data were obtained in the chronic epoch. Each patient's lesion was reconstructed in 3 dimensions using Brainvox software (Damasio and Frank 1992; Frank et al. 1997) and the MAP-3 technique (Damasio et al. 2004). Lesioned tissue evident on each patient's MR or CT scan was manually warped to corresponding anatomy of a normal template brain. The template brain was then used as a common frame of reference to determine the overlap of lesion location among patients. Additionally, the template brain was parcellated according to gyral boundaries (cf. Desikan et al. 2006), which permitted volumetric analysis of the lesions within parcels (Fig. 1 and see Supplementary Fig. 2). One vmPFC participant (ID 10 in Supplementary Table 1) was excluded from volumetric analysis. Although CT data revealed an orbitofrontal/ventromedial lesion in this participant, there was insufficient anatomical detail for application of the MAP3 lesion mapping method. Notably, all analyses of behavioral data were conducted with and without this participant, and the same patterns of vmPFC performance were uniformly observed.

Patients in the vmPFC group were selected on the basis of having damage that included vmPFC in one or both hemispheres, where vmPFC was defined in the space of the template brain and included gyrus rectus, ventromedial superior frontal gyrus (vmSFG), and the medial orbitofrontal gyrus (mOrbG). Lesions were caused by meningioma resection (5), arteriovenous malformation resection (1), subarachnoid hemorrhage (3), and stroke (2). All patients with etiologies of stroke or subarachnoid hemorrhage had involvement of the anterior communicating or anterior cerebral artery and had surgically implanted clips, as did one resection patient.

Volumetrically (see Supplementary Tables 3 and 4), 9 of the 10 patients included in the analysis had lesions that included gray matter within the gyrus rectus bilaterally, and one patient's lesion did not include gray matter of the gyrus rectus (ID 5, refer to Supplementary Tables 1 and 2). Among patients with gyrus rectus damage, the proportion of parcel voxels included in the lesion was similar in the left (mean = 0.550; SD = 0.228) and right (mean = 0.585; SD = 0.288) hemispheres. Meanwhile, 9 of the 10 patients had bilateral lesions that included gray matter in mOrbG, whereas one patient's lesion included only left mOrbG gray matter (5). Among patients with mOrbG damage, the proportion of parcel voxels included in the lesion was somewhat greater on the right (mean = 0.357; SD = 0.281) than on the left (mean = 0.248; SD = 0.181), but the difference was not statistically reliable [*T*_(17)_ = 1.015, *P* = 0.324]. Medially and more superior, 8 of the 10 patients had lesions that bilaterally included gray matter in the vmSFG, while one patient had vmSFG damage limited to the right hemisphere (ID 4), and one had vmSFG damage limited to the left hemisphere (ID 5). Among patients with vmSFG damage, the proportion of parcel voxels included in the lesion was somewhat greater on the right (mean = 0.641, SD = 0.279) than on the left (mean = 0.519, SD = 0.284), but the difference was not reliable [*T*_(16)_ = 0.918, *P* = 0.372]. Finally, a region just outside of what is typically considered human vmPFC is presented for comparison. Only 4 of the 10 patients included in the analysis had lesions that bilaterally included gray matter in the lateral orbitofrontal gyrus (lOrbG), whereas an additional 3 patients had damage to limited to the right lOrbG, and 1 patient had damage limited to the left lOrbG. Among patients with any nonzero lOrbG damage, the proportion of parcel voxels included in the lesion was somewhat greater on the right (mean = 0.216, SD = 0.196) than on the left (mean = 0.075, SD = 0.046), but the difference was not reliable [*T*_(10)_ = 1.55, *P* = 0.151].

#### BDC Group

Participants in the BDC group underwent scanning and analysis procedures identical to those described for the vmPFC group, and neuroimaging data were available for all BDC participants (10 MR and 1 CT). None had damage to any portion of the vmPFC per our definition (see above). Lesions were caused by meningioma resection (*N* = 1), temporal lobectomy (*N* = 2: 1 left and 1 right), arteriovenous malformation resection (*N* = 1), and stroke (*N* = 5). Additionally, two BDC participants had combined etiologies: BDC 8 underwent a right temporal lobectomy and suffered a contemporaneous right anterior choroidal artery stroke; and BDC 11 suffered a combined subarachnoid hemorrhage and infarct. For all BDCs, damage was principally limited to the temporal, occipital, or parietal lobes (see Supplementary Fig. 1).

#### Unique Dorsal Prefrontal Patient

The dPFC participant underwent a bilateral frontal meningioma excision. The extent of the participant's brain injury was assessed using MR data. Significant involvement of gray and white matter in the dorsal medial and lateral prefrontal cortex was evident (see Supplementary Fig. 3), but the vmPFC was preserved.

### Experimental Tasks

Participants were paid $25 for the 2-h test session. Participants were instructed that they would be playing a game in which they would make choices, with their goal being to win as many points as possible. The following versions of the task were used, with the same task order used in all participants tested. No response deadline was used, with participants encouraged to respond as soon as they felt confident of their choice.

#### Probabilistic Task

Participants completed 4 phases of the probabilistic version of the task including phases with only observational learning, only experiential learning, or a mixture of both (Fig. 2). The structure of phase 2 (experiential learning only) was based on the task used previously in Hornak et al. (2004) and O'Doherty et al. (2001). In each experimental phase, different fractal pairs were used. The value of the good stimulus was probabilistically drawn from 1 of 2 uniform distributions: 70% (+80 to +250) or 30% (−10 to −60). The value of the bad stimulus was likewise probabilistically drawn from 1 of 2 different uniform distributions: 60% (−250 to −600) or 40% (+30 to +65). The values of the 2 fractals were generated randomly for each phase and participant: Different pairs of fractals were used in each phase, and on each trial the position (i.e., left or right) of a fractal was randomly generated. In all phases, cumulative totals of participants' overall winnings were presented after every 5 trials. However, trial-by-trial feedback concerning the outcome of participants' choices was only presented in phases 1, 2, and 3 (see below).

##### Phase 1 (experiential learning)

Participants completed “ACTIVE” trials where they received trial-by-trial outcomes related to their choices, with participants performing the task until a criterion of 14 of 16 choices of the good stimulus was reached. Good and bad item value distributions for this phase were different from those used in phases 2–4, as per Hornak et al. (2004). The value of the good stimulus was probabilistically drawn from 1 of 2 uniform distributions: 70% (+60 to +200) or 30% (+10 to +50). The value of the bad stimulus was likewise probabilistically drawn from 1 of 2 different uniform distributions: 60% (−70 to −300) or 40% (+10 to +100).

##### Phase 2 (experiential learning)

Participants completed ACTIVE trials and received trial-by-trial outcomes related to their choices, with a reversal occurring after a criterion of 9 of 10 choices of the good stimulus. The phase was terminated after 70 trials in total.

##### Phase 3 (experiential and observational learning)

Participants performed the task as in phase 2, and received trial-by-trial feedback on ACTIVE trials as to the outcomes of their choices. However, on alternate “WATCH” trials, participants did not make choices themselves, but instead were given the opportunity to observe the choices and outcomes generated by a computer player. While participants were aware that the computer's actual choices were randomly determined and that they would not receive any rewards for the computer's choices, they were instructed that the computer was effectively choosing in the same environment (i.e., with the same underlying reward schedules), and therefore they could learn about the values of the fractals through observation. Participants were also told that they would not win or lose points during WATCH trials, and running totals of accumulated points (presented after every fifth choice by the participant) did not reflect or report computer wins or losses. This phase totaled 70 ACTIVE and 70 WATCH trials. Reversals occurred as described for phase 2.

##### Phase 4 (observational learning only, reversal component, outcomes displayed only for computer choices, and not for participant choices)

This phase was identical in format to phase 3 (i.e., alternating ACTIVE and WATCH trials), with the notable exception that participants did not receive feedback concerning the reward outcomes of their own choices during ACTIVE trials. Thus, participants could only learn the stimulus values through observation, by watching the computer's choices and outcomes. Cumulative point totals were provided after every fifth participant choice. Reversals occurred as described for phase 2.

#### Deterministic Task (Experiential Learning)

This paradigm generally followed the procedure used by Fellows and Farah (2003) and was used as a control task to measure experiential learning performance in a very simple context. In our implementation, participants were required to choose between 2 fractals, which we refer to as A and B, presented on the left and right sides, respectively, of the display; position was randomized. On any given trial, one fractal consistently (i.e., with 100% contingency) yielded a gain of 50 points (“good” stimulus), with the other fractal yielding a loss of 50 points (“bad” stimulus). Trial-by-trial feedback was provided to the participants, in addition to a display of their cumulative point total after every 5 trials. Reversals occurred as described for phase 2 of the probabilistic task. Each participant completed 70 trials in total.

### Analytic Procedures

In the probabilistic task, the reward values of the 2 fractals were randomly generated for each phase and participants according to the prespecified schedule described above. This was done to avoid the possibility that observed deficits could arise due to the specific properties of any particular reward schedule (i.e., a possible concern with the use of a single reward schedule across all participants). To control for possible differences in average reward values of stimuli between participant groups, we computed the total number of points won in any phase above that predicted by random choice (which would be equivalent to the average value of both stimuli over trials). This measure (“points won”) is used throughout and entered into the statistical analyses performed. Basic statistical analyses were performed in SPSS 19 and R software. Statistical comparison of the single dPFC patient with the performance of the other experimental groups (control, vmPFC, and BDC) was performed using the modified Crawford's *t*-test which was designed for this purpose (Crawford and Garthwaite 2002).

#### Logistic Regression

We performed an analysis to examine the influence of previous outcomes (i.e., points won) and choices on current behavior. To achieve this, we carried out a logistic regression analysis following the procedure previously used to evaluate the performance of monkeys (Lau and Glimcher 2005; Kennerley et al. 2006) and humans (Rutledge et al. 2009; Kovach et al. 2012). This procedure has the advantage of allowing choice parameters to be included in the model in order to capture the effect of the past history of choices, a feature which is particularly relevant in the current context given previous reports that patients with vmPFC damage may exhibit reversal deficits due to an increased tendency to perseverate (McEnaney and Butter 1969; Rolls et al. 1994; Fellows and Farah 2003).

This analysis seeks to estimate weights (i.e., coefficients), which define the contribution of past rewards and choices to current choice, indexed by the choice log odds.

$$\log\frac{P_{A,\;t}}{P_{B,\;t}}=\sum_{\;j=1}^{5}\alpha_{\;j\;}(R_{A,\;t-j}-R_{B,\;t-j})+\sum_{\;j=1}^{5}\beta_{\;j\;}(C_{A,\;t-j}-C_{B,\;t-j})+\gamma$$

where *A* = fractal A, *B* = fractal B; $\log P_{A,\;t}/P_{B,\;t}$ is the choice log odds; *t* is the current trial; $\alpha_{\;j\;}$ is a reward coefficient relating to *j* trials in the past; $\beta_{\;j\;}$ is a choice coefficient relating to *j* trials in the past; $R_{A,\;t-j}$ is the magnitude of reinforcement (i.e., points won) received for choosing fractal A at *j* trials in the past; $C_{A,t-j}$ is 1 if fractal A was chosen at *j* trials in the past (and zero otherwise). γ is an intercept term that captures any residual bias not explained by past rewards and choices. We included both subject choices and rewards received, and those of a computer player (where relevant, i.e., phases 3 and 4), in the regression model, with the aim of capturing the influence of both directly experienced, and observed, actions and outcomes on current behavior. Given that subjects did not receive feedback relating to their choices in phase 4, the magnitude of subject rewards for phase 4 was not included (i.e., coded as zero). Subject choices were entered into the regression analysis in all phases.

Positive weights, therefore, index an increase in the log odds (i.e., a tendency to favor fractal A), as a function of past rewards $\left(\alpha_{\;j\;}\right)$ or choices $\left(\beta_{\;j\;}\right)$. Given that in our study subjects had to choose between 2 options in our task, we assume symmetrical weights for both options [as in Lau and Glimcher (2005)]: that is, a reward obtained *j* trials ago increases the log odds by $\alpha_{\;j\;}$ if it was received through choosing fractal A, but decreases the log odds by $\alpha_{\;j\;}$ if it was received by choosing fractal B.

For example, an $\alpha_{2}$ of 0.002 indicates that a positive reward of 200 points received by choosing fractal A 2 trials in the past would increase the choice log odds by 0.4 (or the odds by ${\text{e}}^{0.4}=1.49$).

$$\log\frac{P_{A,\;t}}{P_{B,\;t}}R_{A,\;\;t-j}C_{A,\;\;t-j}{\text{e}}^{0.4}=1.49.$$

#### Model Comparison

Following Lau and Glimcher (2005), we carried out a search of the parameter space, considering models that included differing trial length histories relating to human and computer player choices and reward outcomes. To restrict the overall number of parameters given the size of our experimental dataset and ensure the robustness of fit, we constrained our search to 6 ACTIVE trials in the past. Given that WATCH trials alternated with ACTIVE trials in phases that incorporated observational learning (i.e., phases 3 and 4), we considered models that included terms for up to 3 WATCH trials in the past (i.e., since if the participant was currently on a WATCH trial, the *t*−1 WATCH trial actually occurred 2 trials ago due to the intervening ACTIVE trial).

As in previous work (e.g., Lau and Glimcher 2005; Daw et al. 2006; Daw 2011), models were fit using a process of maximum likelihood estimation. We computed the fit of the regression model using a pseudo-*R*^2^ statistic:

$$\text{Pseudo - }R^{2}=\frac{\left(\text{L}{\text{L}}_{R}-\text{L}{\text{L}}_{M}\right)}{\text{L}{\text{L}}_{R}}.$$

where LL_R_ indexes the maximum log likelihood of the model under random choice, and LL_M_ indexes the log likelihood of the estimated model given the data. Regression models with different trial histories (e.g., 5 trials in the past vs. 3 trials) were compared using a standard measure, that is, the Akaike information criterion (AIC).

$$\text{AIC}=-2\;\text{logL}{\text{L}}_{M}+2k,$$

where LL_M_ is the maximum log likelihood of the estimated model, and *k* is the number of parameters. Models with lower AIC values are preferred, where a difference of >10 indicates strong support for the better fitting model.

The main regression model reported (see Results) included terms representing: (1) rewards received by the participant after each of the previous 5 trials; (2) choices made by the participant during each of the previous 5 trials; (3) rewards received by the computer player (in phases 3 and 4) after each of the previous 3 computer trials; and (4) choices made by the computer player (in phases 3 and 4) on each of the previous 3 computer trials. The model specified above, consisting of 16 parameters and a constant term, provided the best observed fit to the overall dataset, indexed by the pseudo-*R*^2^ measure (0.26); and an AIC of 2391, which takes into account model complexity. In comparison, simpler models (e.g., modeling the effect of only 1 past trial for both human and computer rewards and choices) provided a substantially worse fit to the data: AIC = 2461.

#### Parameter Evaluation

To avoid assumptions of normality and symmetry in our parameter estimates, we evaluated differences between parameter estimates using nonparametric confidence intervals and *P*-values calculated with the BC_a_ method (DiCiccio and Effron 1996). A total of 1000 bootstrap samples were drawn from our observed data using the methodology of Hsu et al. (2005), which stipulates randomly drawing participants (*N* = 11 in our case) from the original sample with replacement, and forming a single bootstrap sample composed of all observations associated with the resulting sample of participants (Hsu et al. 2005). Parameter values for the best-fit logistic model were recorded for each bootstrap sample. 95% BC_a_ confidence intervals were calculated from these sets of bootstrapped parameters, and the *P-*value reflecting the probability that the interval contained 0 ($P_{BC_{a}}$) was used to evaluate parameter significance. We used this procedure within the comparison and vmPFC groups, and additionally in a combined model including interaction terms for group membership with each parameter.

## Results

Participants completed 4 phases of a probabilistic two-arm bandit task, which differed in terms of whether reversal occurred (phases 2, 3, and 4), and in terms of trial composition (see Fig. 2 and Materials and Methods). As is often the case in neuropsychological studies, patients were reimbursed with a fixed monetary account at the end of the experiment (i.e., rather than points won translating into real monetary gain). During “ACTIVE” trials (phases 1–4), participants selected an option, and in all phases except phase 4, they received trial-by-trial feedback concerning the outcome of their choice. During “WATCH” trials (phases 3 and 4), participants did not make choices themselves but instead were given the opportunity to observe the consequences (i.e., reward outcomes) of externally generated actions (i.e., of the computer player). Different experimental phases, therefore, involved either only experiential learning (phases 1, 2), only observational learning (phase 4), or a mixture of both (phase 3).

### Overall Performance on Probabilistic Task

We first considered participants' performance across the 4 experimental phases in terms of overall aggregate measures, such as the total number of points won and tendency to switch after a high magnitude loss. The pattern of performance was generally similar for all groups, who earned approximately the same number of points per condition. The only exception was in the observational-only condition (phase 4), in which vmPFC patients earned fewer points than comparisons prior to their first reversal. To anticipate, this modest deficit may reflect the more significant deficit that we observed in our subsequent regression analysis (see the next section).

In phase 1 (experiential learning only: Materials and Methods and Fig. 2), the vmPFC group required 45 trials on average to reach a criterion (SEM = 11.3; see Supplementary Table 5), defined as the choice of the higher valued stimulus on 14 of the 16 preceding trials, whereas NCs averaged 33 trials to reach a criterion (SEM = 6.2) and BDCs averaged 31 trials to reach a criterion (SEM = 5.2). No significant group-level differences were found for this measure or for points won (each *P* > 0.2; Fig. 3). A repeated-measures ANOVA confirmed that the choice behavior of all groups showed was sensitive to large magnitude trial outcomes, indexed by a significantly greater tendency to switch to the alternative stimulus following a large magnitude loss (i.e., >200 points) when compared with a large magnitude win (i.e., >100 points): main effect of the outcome valence—*F*_1,27_ = 20.457, *P* < 0.0005; no significant group × valence interaction, *F*_2,27_ = 0.324, *P* > 0.7. No significant difference was found in overall switching tendency between groups (*F*_2,27_ = 1.387, *P* > 0.25).

We next examined the overall performance of participants in experimental phase 2 (experiential learning only) and 3 (experiential and observational learning; Fig. 3 and see Supplementary Table 5). No significant differences were observed between the participant groups along any measure of aggregate performance (*P* > 0.1). All participant groups, therefore, were able to modify their behavior in response to big wins and big losses (both as defined earlier): main effect of valence, each *F*_1,30_ > 50, each *P* < 0.0001; no significant interactions, each *P* > 0.5. Furthermore, there were no significant differences between participant groups in cumulative gain (i.e., points won) over the course of the experiment (each *F*_2,30_ < 0.4, each *P* > 0.7) or the number of reversals achieved (each *F*_2,30_ < 0.9, each *P* > 0.4). Interestingly, therefore, the performance of our cohort of vmPFC patients based on directly experienced actions and outcomes (i.e., phase 2) was indistinguishable from that of both NC and BDC participants. Given prior work suggesting that lesions of the lateral orbitofrontal cortex (OFC)—or combined lesions of the lateral and medial OFC (Rudebeck and Murray 2011b)—rather than the vmPFC, produce marked reversal learning deficits (Rushworth et al. 2011), we asked whether damage to this brain region predicted performance. No significant correlations were found between performance in any phase (i.e., either in terms of points won or number of reversals) of individual vmPFC patients and the extent of damage to the lateral orbital gyrus (each *P* > 0.1), as measured using volumetric analyses (see Supplementary Table 4 and Materials and Methods). Note, however, that this analysis can be considered exploratory and underpowered given the relatively limited extent of damage to those regions in most patients (<10% overall, many with no damage or no damage: see Fig. 1 and Supplementary Fig. 1 and Table 4).

We next considered the overall performance of the vmPFC patient cohort in phase 4 (observational learning only), where learning was dependent solely on the observation of reward outcomes associated with the actions of a computer player during WATCH trials (i.e., participant choices during ACTIVE trials were not followed by feedback, although the unseen reward was counted toward their cumulative total points won as in other phases), which was known to be playing in the same environment but whose choices were random. Interestingly, all participant groups demonstrated a substantial capacity to learn about the values of stimuli, and to update these estimates in the face of change (i.e., reversals), through observation. All participants gained significantly more points than predicted by chance (each *t*_(10)_ > 2.5, each *P* < 0.05): vmPFC (mean points won: 3486 [1350]); NC (mean points won: 4127 [891]); BDC (mean points won: 4528 [770]; see Fig. 3 and Supplementary Table 5). While all groups performed similarly in this phase according to several measures including the number of reversals, points won after the first reversal, and switching behavior (all *P* > 0.1), both comparison groups outperformed vmPFC participants in the number of points won prior to the first reversal, *F*_2,25_ = 6.9, *P* = 0.004 (vmPFC mean = 510 [339], NC mean = 1529 [267], and BDC mean = 1908 [219]). Planned comparisons between vmPFC and NC groups (*t*_(16)_ = 2.361, *P* = 0.031) and vmPFC and BDC groups (*t*_(17)_ = 3.5, *P* = 0.003) indicated that these differences were significant; meanwhile, the comparison groups did not differ (*t*_(17)_ = 1.1, *P* > 0.2). While vmPFC participants also required numerically more trials to trigger the first reversal in phase 4 (vmPFC mean = 24.8 [5.3], NC mean = 16.3 [4.0], and BDC mean = 19.1 [2.1]), this difference did not approach significance (*F*_2,25_ = 1.2, *P* > 0.3).

### Logistic Regression Analyses: Experiential Learning

The relatively intact performance of vmPFC patients indicates that generally normal experiential learning is possible in the face of vmPFC damage. Critically, however, measures which summarize performance across an entire phase (e.g., total points won) may be insensitive to subtle abnormalities in underlying value representations. Therefore, we applied a finer-grained method of analysis to characterize the choice data using a logistic regression model (cf. Lau and Glimcher 2005; Rutledge et al. 2009; Kovach et al. 2012; see Materials and Methods). Importantly, our study generated a large quantity of choice data consisting of several thousand trials per group, making it well suited to this type of analysis.

Logistic regression analyses afford the opportunity to examine the influence of past rewards and actions on current behavior, without assuming that these influences decay exponentially over time (i.e., as in reinforcement learning models; Lau and Glimcher 2005; Rutledge et al. 2009). This procedure seeks to estimate weights (i.e., coefficients), which define the contribution of past rewards and choices to current choice, up to *j* trials in the past (see Materials and Methods). While the reward weights ($\alpha_{\;j\;}$) index the influence of a reward at trial *t*−*j* in the past on current choice at trial *t*, the choice weights $(\beta_{\;j\;})\;$ index the tendency of a given participant to perseverate (i.e., repeat the same choice) independent of any association of choice with reward. Separate coefficients were estimated for both directly experienced choices and outcomes (i.e., relating to the participants themselves during ACTIVE trials) and observed choices and outcomes (i.e., of the computer player during WATCH trials, see Materials and Methods). Positive weights, therefore, index an increase in the choice log odds (e.g., tendency to favor fractal A), as a function of past rewards ($\alpha_{\;j\;}$) or choices $(\beta_{\;j\;})\;$.

Data from all experimental phases of the probabilistic task were entered into the regression model to increase the size of the dataset and reliability of the parameter estimates (Lau and Glimcher 2005). The best-fitting regression model (see Materials and Methods for details of fitting procedure) included terms representing: (1) Rewards received by the participant during each of the previous 5 ACTIVE trials (in phases 1, 2, and 3); (2) choices made by the participant during each of the previous 5 ACTIVE trials; (3) rewards received by the computer player (in phases 3 and 4) during each of the previous 3 WATCH trials; and (4) choices made by the computer player (in phases 3 and 4) on each of the previous 3 WATCH trials (Materials and Methods).

The results of an overall ANOVA which included factors comprising participant group, latency (i.e., trials into the past), and information type (i.e., directly experienced and observed choices and reward outcomes) are summarized in Supplementary Table 6. There was a significant effect of group [*Χ*^2^(50) = 72.02, *P* = 0.022], and to anticipate the results, this was driven by a specific deficit in observational learning in the vmPFC group (see below). We first consider effects relating to experiential learning—that is, the influence of participants' own past choices and reward outcomes on current behavior.

**Figure 4.**
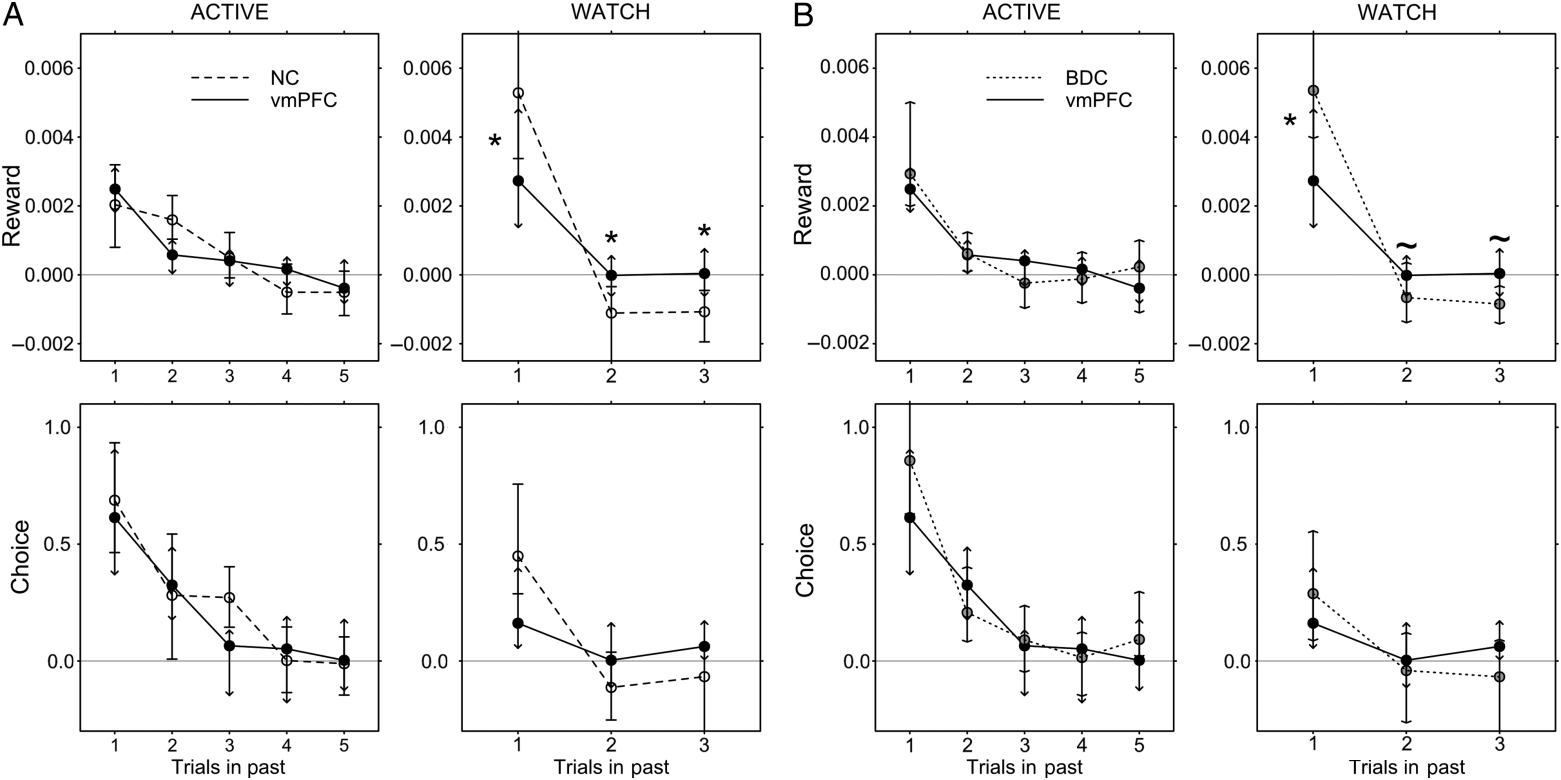
Results of logistic regression: Influence of previously experienced and observed rewards and choices on current behavior. (*A*) vmPFC and NC groups and (*B*) vmPFC and BDC groups. Experiential learning (“ACTIVE” trials: left-side panels): Top panels show influence of past rewards and bottom panels show influence of past participant choices on current behavior. *x*-axis refers to the number of trials in the past (e.g., Rewards: from the previous trial, *t*−1, until 5 trials in the past, *t*−5). Regression coefficients, for rewards (top panels) or choices (bottom panels), plotted on *y*-axis (solid line indicates the vmPFC group, and dashed line indicates the appropriate comparison group group). For example, a reward coefficient of 0.003 relating to the *t*−1 trial indicates that the receipt of +100 points for choosing fractal A on the previous trial would increase the choice log odds of choosing fractal A on the current trial by 0.3 (i.e., 0.003 × 100). Similarly, a choice weight of 0.6 relating to the *t*−1 trial indicates that choosing fractal A on the previous trial would increase the log odds of selecting fractal A on the current trial by 0.6, above and beyond any reward received on the last trial (or, indeed, any rewards or choices relating to the computer player). See Materials and Methods for details. Observational learning (“WATCH” trials: right-side panels): Top panels show the influence of past observed rewards and bottom panels show the influence of past observed choices on current behavior. *x*-axis refers to the number of trials in the past (from the previous, *t*−1, trial until 3 trials in the past, *t*−3). Regression coefficients, for rewards (upper) or choices (lower panels), plotted on *y*-axis (solid line indicates the vmPFC group, and dashed line indicates the appropriate comparison group). For example, a reward coefficient of 0.004 relating to the *t*−1 trial indicates that if the “computer player” received a loss of −500 points for choosing fractal A on the previous WATCH trial, this would decrease the log odds of “the participant” choosing fractal A on the current ACTIVE trial by 2 (i.e., 0.004 × 500). Similarly, a choice weight of 0.4 relating to the *t*−1 trial indicates that if the computer player had chosen fractal A on the last WATCH trial, this would increase the log odds of the “participant” selecting fractal A on the current ACTIVE trial by 0.4, above and beyond any reward received by the computer on the last WATCH trial (and, indeed, any rewards received by the participant, or choices made by the participant). See Materials and Methods for details. Whiskers indicate BC_a_ 95% confidence intervals [CIs; see DiCiccio and Effron (1996)]: Right-angle endlines are associated with CIs for comparison participants, and angled endlines with CIs for vmPFC participants. Note that overlapping BC_a_ confidence intervals do not indicate nonsignificant differences; a better indicator of group differences is exclusion of the contrasting mean value from the interval. Symbols: *$P_{BC_{a}}<0.05$; ^∼^$P_{BC_{a}}<0.10$.

As expected, there was a significant effect of participant rewards during ACTIVE trials [*Χ*^2^(15) = 1217.10, *P* < 0.0001: Fig. 4], whose influence decayed with increasing time into the past [latency × participant reward interaction: *Χ*^2^(12) = 25.76, *P* = 0.012]. There were no significant interactions of these factors with the group [*Χ*^2^(8) = 8.16, *P* = 0.418]. In NC participants, the influence of rewards for trials *t*−1 ($P_{BC_{a}}<0.0001$, where $P_{BC_{a}}$ values were calculated using the BC_a_ method and nonparametric confidence intervals—see Materials and Methods), *t*−2 ($P_{BC_{a}}<0.0001$), and *t*−3 ($P_{BC_{a}}<0.05$) was significant, whereas that at trial *t*−4 was marginal ($P_{BC_{a}}=0.11$). The vmPFC patients also showed effects of recent rewards on current choices at trials *t*−1 ($P_{BC_{a}}<0.0001$), *t*−2 ($P_{BC_{a}}<0.05$), and *t*−3 ($P_{BC_{a}}<0.05$). While pairwise comparisons suggested that the influence of rewards received at trial *t*−2 was reduced in both the vmPFC and BDC groups relative to the NC group ($P_{BC_{a}}<0.005\;\text{and}\;<0.05,$ respectively), these effects cannot be considered reliable in the absence of a Group × Participant Reward × Latency interaction in the overall ANOVA (see Supplementary Table 6).

We also found a significant effect of past participant choices during ACTIVE trials on behavior [*Χ*^2^(15) = 1365.71, *P* < 0.0001], with the influence of past choices decaying with increasing latency [latency × participant choice interaction: *Χ*^2^(12) = 378.60, *P* < 0.0001]. No interaction of these factors with group was found [*Χ*^2^(8) = 7.50, *P* = 0.484]. On any given trial, therefore, all participant groups tended to repeat choices they had made in the past—an effect that extended in NC participants until trial *t*−3 (each time point, $P_{BC_{a}}<0.001$), and in vmPFC and BDC patients to trial *t*−2 ($P_{BC_{a}}<0.005$). Interestingly, this tendency to repeat past choices independent of any rewards received (i.e., perseverate) was in fact most prominent in the NC group: The choice made in trial *t*−3 exerted a significantly greater influence on the current choice in NC participants than in vmPFC patients ($P_{BC_{a}}<0.05$), with a marginally significant effect observed when the NC group was compared with the BDC group ($P_{BC_{a}}=0.058$). Notably, however, the absence of a significant Group × Choice × Latency effect in the overall ANOVA [*Χ*^2^(8) = 7.50, *P* = 0.484] suggests caution in interpreting this perseverative tendency in NC participants.

#### Results: No Evidence for a Failure of Contingent Learning in Patients with vmPFC Damage

**Figure 5.**
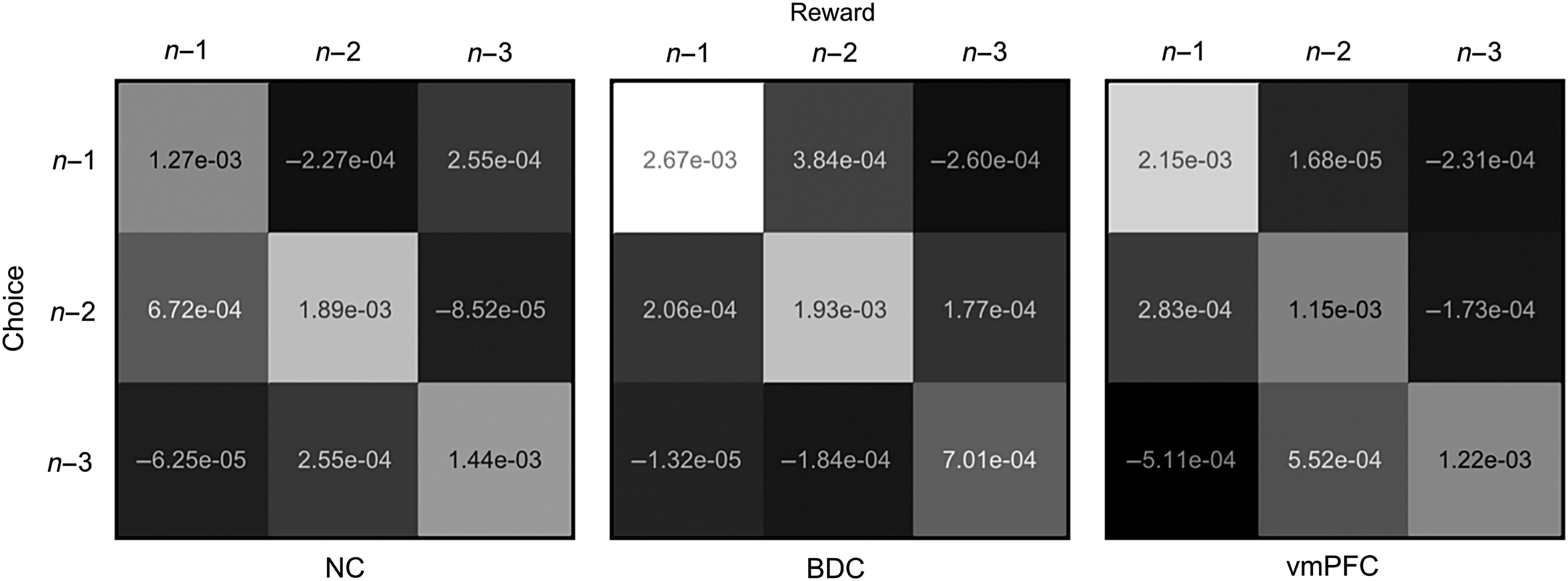
Results of logistic regression analysis designed to assess contingent learning. Contingent learning refers to the formation of discrete associations between an individual choice (e.g., on the last, *n*−1, trial) and its specific outcome (i.e., on the *n*−1 trial), as opposed to false associations between different trials (e.g., *n*−2) and other outcomes (e.g., *n*−3 trial). The matrices (left: NC, center: BDC, and right: vmPFC group) show the magnitude of regression coefficients (white = large and black = small) relating to the association of each of the 3 past reward outcomes with each of the past 3 choices. Squares on the diagonal, therefore, relate to contingent learning: that is, veridical associations between a specific trial (e.g., *n*−1) and the specific outcome received (at *n*−1 trial). Off-diagonal components index the strength of false associations: for example, the bottom left square relates to the association of the reward received on the last trial (*n*−1) with the choice made on the *n*−3 trial. As such, a high magnitude coefficient indexed by this component would indicate that if fractal A had been chosen 3 trials in the past, then receiving a large reward on the last trial would increase the log odds of selecting fractal A on the next trial, irrespective of which fractal was chosen on the *n*−1 trial and what reward was received on the *n*−3 trial. See the main text for details.

Our findings, therefore, suggest that our cohort of vmPFC patients retain a similar capacity as the BDC group in adapting their behavior based on the rewarding consequences of their own internally generated choices, with only equivocal evidence that this differed from that of the NC group. We next considered whether damage to the vmPFC might specifically impair “contingent learning,” whereby discrete associations are formed between individual choices on a given ACTIVE trial and their specific outcomes (Walton et al. 2010; Rushworth et al. 2011). Indeed, recent work in monkeys suggests that the lateral OFC may play an important role in maintaining a neural representation of current choice and the outcome, facilitating their association with one another (Walton et al. 2010). As such, damage to the lateral OFC in non-human primates results in a phenomenon known as a “spread of effect,” whereby reinforcement information on a given trial is assigned backwards, or even forwards, in time, therefore becoming associated with past or future actions (Walton et al. 2010). Blurring of the specific choice–outcome history in this way can lead to surprising consequences: for example, the receipt of a large loss when a given stimulus (e.g., fractal A) was chosen on trial *t*−1 might aberrantly increase the probability of reselecting this stimulus on the current trial (*t*), if the same stimulus had been associated with a favorable outcome on previous trials (e.g., the *t*−2 trial). In our next analysis, therefore, we asked whether our cohort of vmPFC patients might have a specific deficit in contingent learning. To carry out this regression analysis, we included all possible combinations of associations between choices and outcomes pertaining to the last 3 trials in the regression model, following the procedure of Walton et al. (2010). We found no evidence for a failure of contingent learning in patients with vmPFC damage. Specifically, the influence of specific choice–outcome associations (e.g., *t*−1 reward with choice on *t*−1 trial) was greater than false associations (e.g., reward received on trial *t*−1 with choice made on trial *t*−2) in vmPFC patients, NC participants, and BDC patients (Fig. 5).

### Logistic Regression Analyses: Observational Learning

We next examined the capacity of vmPFC patients, and the 2 comparison groups, to learn in an observational fashion, in phases 3 and 4 where participants had the opportunity to benefit from viewing the choices and outcomes of a computer player on alternate WATCH trials (see Materials and Methods). As stated previously, participants were instructed that the computer's choices were entirely random, but also that they would be able to learn through observation of the resultant outcomes. Participants' current choices were significantly influenced by the observed outcomes relating to the computer player [*Χ*^2^(15) = 1082.78, *P* < 0.001]. There was a positive influence of past computer rewards relating to the immediately preceding trial (i.e., *t*−1) in all groups (reward weights: *P* < 0.0001 all groups), which declined over time into the past [latency × computer reward interaction: *Χ*^2^(12) = 236.73, *P* < 0.001]. However, there was a specific deficit in the vmPFC group in using previously observed reward outcomes to guide their choice behavior, evidenced by a significant group × computer reward interaction [*Χ*^2^(10) = 42.34, *P* < 0.0001; Fig. 4 and see Supplementary Table 6]. Pairwise comparisons revealed that both NC and BDC groups showed a significantly greater influence of *t*−1 rewards than vmPFC patients (NC $P_{BC_{a}}=0.033$ and BDC $P_{BC_{a}}=0.016$). The influence of observed rewards did not differ between NC and BDC groups ($P_{BC_{a}}>0.1$). Of note, there was also a significant group × latency × computer reward interaction [*Χ*^2^(8) = 41.05, *P* < 0.0001]: This was driven by a tendency for the NC and BDC groups to select the less rewarding of the 2 stimuli observed on WATCH trials occurring at *t*−2 and *t*−3 trials in the past (see Fig. 4A and B, top right panels), in contrast to the vMPFC group where *t*−2 and *t*−3 trial outcomes exerted no influence on current choice (i.e., regression coefficient = 0). While it is difficult to provide a definitive interpretation of this finding—which is qualitatively mirrored in the analysis of observed choices (see Fig. 4A and B bottom right panels)—it is consistent with the overall diminished influence of previously observed outcomes on current choice in the vmPFC group, and we speculate that this may reflect strategic biases in the NC and BDC groups.

We also found that participants' current choices on ACTIVE trials were significantly influenced by the past choices made by the computer player during WATCH trials [*Χ*^2^(15) = 97.30, *P* < 0.001]. As such, all groups had a tendency to follow the previous action of the computer player, even though this was known to be random in nature, and therefore uninformative. This influence also decreased over time into the past [latency × computer choice interaction: *Χ*^2^(12) = 41.51, *P* < 0.001; see Supplementary Table 6]. As such, the influence of observed choices also extended only to the immediately preceding trial (i.e., *t*−1 choice weights, NC group, $P_{BC_{a}}<0.001$; vmPFC group $P_{BC_{a}}=0.005$; BDC group $P_{BC_{a}}=0.010$; Fig. 4b). Interestingly, the tendency of the NC group to repeat the computer player's last action was significantly greater than that of the vmPFC group ($P_{BC_{a}}=0.010$), whose performance was not distinguishable from that of the BDC group ($P_{BC_{a}}>0.2$). However, this choice-related finding should be considered unreliable, given the absence of a significant group × computer choice effect [*Χ*^2^(10) = 13.47, *P* = 0.1985].

Our findings provide evidence that the vmPFC makes a specific contribution to the learning of values through the observation of the reward outcomes that resulted from externally generated actions (i.e., of a computer player) during WATCH trials. Given previous work implicating the vmPFC in reversal learning (e.g., Fellows and Farah 2003), however, we next considered the possibility that this deficit in observational learning might reflect an impairment in updating values after a reversal has occurred. To consider this possibility, we restricted the logistic regression analysis to trials before the first reversal had occurred, in each experimental phase. Given the reduced quantity of data entered into the model (approximately 1200 trials per participant group), we adjusted the number of parameters to increase the robustness of our model's fit, and so the influence of participant and computer choices and rewards was restricted to *t*−3 trials in the past. Importantly, a qualitatively similar pattern of findings was observed to those reported for the full dataset. Specifically, we found a selective deficit in the ability of vmPFC patients to use observed rewards to guide their decision-making behavior [three-way interaction of group by latency by computer reward, *Χ*^2^(4) = 18.63, *P* < 0.001]. This result, therefore, confirms that patients with vmPFC damage show a reduced influence of observational rewards on current behavior during initial learning relative to the comparison groups, even prior to the occurrence of any reversals.

As a supplemental analysis, we also included the cumulative point totals that were presented every fifth trial to participants (see Materials and Methods) as an additional variable in the regression analysis relating to phase 4. A qualitatively similar pattern of findings was observed as in the primary analysis reported above, with no significant effect of cumulative totals on choice behavior (*P* > 0.1).

We also considered the possibility that patients with vmPFC damage may be impaired at observational learning because it is simply more difficult than experiential learning in our paradigm. To address this issue, we examined the response time (RT) data obtained during ACTIVE trials: We found that the RT of vmPFC patients was numerically [but not statistically; each *T*_(12)_ < 0.5, each *P* > 0.6] faster in the experimental phases that involved observational learning (i.e., phases 3 and 4), when compared with phase 2 which involved purely experiential learning (see Supplementary Fig. 4). Importantly, there was no significant group × phase interaction in terms of RT (*F*_4,60_ = 0.083, *P* = 0.987), arguing against the notion that there was an increase in task difficulty for the vmPFC group in experimental phases involving observational learning (also see Discussion for further consideration of this point).

We also considered the possibility that the impairment in observational learning exhibited by vmPFC patients might be accounted for through a difference in general motivation—specifically, that their apparent deficit might be driven selectively by their performance in phase 4 (i.e., pure observational phase: see Materials and Methods) due to a total absence of directly experienced rewards which led them to be insufficiently motivated. Arguing strongly against this possibility, we found that patients with vmPFC damage showed a deficit in observational learning even when the logistic regression analysis was restricted solely to trials from phase 3 ($P_{BC_{a}}<0.001$), where directly experienced rewards occurred on alternate trials. Moreover, the same pattern was also observed in an analysis using only data from phase 4 ($P_{BC_{a}}<0.001$).

#### Results: Performance of a Patient with Damage to Bilateral Dorsolateral PFC on the Probabilistic Task

In summary, our results point to a specific deficit in observational learning in patients with damage to the vmPFC. In contrast, our results do not provide strong support for the role of the vmPFC in experiential learning, at least in the context of the relatively simple probabilistic scenarios examined (see Discussion). One question that arises is whether this dissociation between experiential and observational learning is specific to the vmPFC or also applies to other PFC regions. While a single neuropsychological study by nature is not able to localize a single cognitive function (e.g., observational learning) to a unique brain region (e.g., vmPFC), we sought additional neuroanatomical constraint by assessing the effects of damage to the dorsal PFC [i.e., sparing vmPFC: see Supplementary Fig. 3 and Table 2 (legend) on performance in our task].

Since lesions affecting the dorsal PFC bilaterally are rare, we examined the single case extant to our knowledge in the Iowa Registry (see Materials and Methods). Due to the dPFC patient's unique status in our study, the direct statistical comparisons used elsewhere to test the performance of other groups against one another were not possible. Instead, we provide specific details about his performance in each condition of each task accompanied where possible by statistical comparisons using the modified Crawford's *t*-test [i.e., designed for use in comparing single cases with groups: see Materials and Methods (Crawford and Garthwaite 2002)]. The difference in performance between the dPFC case and other groups (i.e., vmPFC, NC, BDC) groups was striking (see Supplementary Tables 5 and 7). In terms of coarse aggregate measures (see Supplementary Table 5), the dPFC case failed to reverse in any session, scored below chance performance in all sessions but one (session 3), and showed no sensitivity to large magnitude gains or losses (i.e., indexed by % switching tendency: see Supplementary Table 5). The performance of the dPFC case was significantly worse than that of the other patient groups in nearly all sessions (i.e., using modified Crawford's *t*-test; see Methods and Supplementary Table 7 for details).

Logistic regression analysis was also applied to the dPFC patient's choice and reward history as described above, although it should be noted that given this is a single case relatively few trials were available. Therefore, the fitted parameter values should be interpreted with caution as a result. This analysis suggested that the patient was largely insensitive to the history of rewards or choices, whether his own or that of the computer player (see Supplementary Fig. 5; red dots indicate dPFC patient parameter estimates). This contrasts sharply with the performance of the other groups, who typically showed significant evidence of sensitivity to reward and choice history even, for example, damage to vmPFC was related to reductions in the size of the parameter estimates relating to observational learning.

These findings are consistent with the notion that value signals are distributed across prefrontal regions (Kennerley et al. 2009; Rushworth et al. 2011). While we are cautious about drawing conclusions from a single case study, these results suggest that the dPFC may play a role in feedback-driven learning regardless of whether the feedback is experienced or observed, at least in the context of the experimental setting examined. In relation to this, it is worth bearing in mind that in our paradigm, the magnitude of rewards was informative (cf. other settings where reward magnitude is fixed at +1 or −1, and only the probability varies across stimuli: also see Discussion)—an aspect of the task that may interact with the impairment in executive function (i.e., Wisconsin card sorting task—see Supplementary Table 2) shown by the dPFC case. Regardless, the results from the analysis of this dPFC case support the specific contribution of the vmPFC to observational learning, over and above its contribution to experiential learning.

#### Results: Overall Performance on Deterministic Task

Given that these results were obtained in the context of a probabilistic reward environment, for completeness we also assessed the experiential learning performance of our cohort of vmPFC patients in a deterministic setting. This was motivated by a previous report which found that a separate cohort of patients with damage to the vMPFC shows marked impairments in such a setting (Fellows and Farah 2003). Supplementary Table 8 and Figure 6 illustrate the performance of vmPFC and both comparison groups under these conditions, according to a number of basic measures such as total points won and number of reversals achieved. While no significant group difference was observed between vmPFC patients and comparisons on either of these measures (each *F*_2,30_ < 2.4, each *P* > 0.1), the exceptional nature of the performance of 2 vmPFC patients was noteworthy. The total number of points won by these patients (200 and −100 by patients 8 and 3, respectively) fell more than 6 SD below the mean performance of BDCs (mean = 2082, SD = 282), more than 5 SD below the mean performance of NCs (mean = 2000, SD = 352), and more than 4 standard deviations below the mean of the remaining vmPFC patients (mean = 2106, SD = 397, see Supplementary Fig. 6). Additionally, these 2 patients completed only 1 reversal each, falling more than 7 SD below the average performance of the other vmPFC patients (mean = 4.7 reversals, SD = 0.5), more than 5 SD below the performance of BDCs on this measure (mean 4.3, SD = 0.8), and more than 4 SD below the performance of NCs (mean = 4.6, SD = 0.8).

In contrast to the findings of Fellows and Farah (2003), a substantial majority (i.e., 9/11) of the patients in our cohort performed equivalently to NC and BDC participants on the deterministic version of the reversal task. Despite the low variance in the independence on activities of daily living (IADL) scores of our vmPFC group, we also observed a significant correlation between this measure and performance of patients on the reversal task as indexed by total points won and number of reversals achieved (both *r* > 0.6, *P* < 0.05). Note, however, that this correlation was driven primarily by the marginally low (i.e., 20/21) IADLs of the 2 patients mentioned above who performed very poorly on the reversal task. As reported previously for the probabilistic scenario, no significant correlations were found (*P* > 0.1) between performance (i.e., either in terms of points won or number of reversals) of individual vmPFC patients and the extent of damage to the lateral orbital gyrus, as measured using volumetric analyses (see Materials and Methods).

Notably, the dPFC case performed poorly in this setting also, failing to complete any reversals. Even in this task, the dPFC patient scored only 300 points (see Supplementary Table 8), a performance matched by only the very poorly performing 2 vmPFC patients (see above).

## Discussion

Our neuropsychological study investigated the contribution of the vmPFC to experiential and observational learning—a distinction operationalized here as the ability to update stimulus values based on the rewarding outcomes that follow either internally generated (i.e., experiential) or externally generated (i.e., observational) actions. Fine-grained analysis of experiential choice behavior revealed that the current behavior of vmPFC patients was significantly influenced by past rewards and choices in a manner that was equivalent to a BDC group, and only marginally different from the NC group. Despite this relatively intact capacity for experiential learning, patients with vmPFC damage exhibited a significant deficit in observational learning, manifest in the reduced influence of previously observed rewards on current choices. Our findings provide causal evidence that the vmPFC is necessary for normal learning of stimulus values from observed rewards, and point toward the conclusion that there are dissociable neural circuits for experiential and observational learning.

It is interesting to consider our findings in relation to a previous neuroimaging study by Walton et al. (2004), which used an experimental design that has conceptual similarities with our own (Walton et al. 2004). In their experiment, participants were required to adjust their behavior by monitoring outcomes under 2 different conditions: One in which participants had freely chosen the action themselves and the other in which the action to be performed was instructed by an external cue specified by the experimenter. They reported a dissociation between the medial and lateral OFC and the dorsal anterior cingulate cortex (ACC): Neural activity in the OFC was greatest when participants were required to monitor the consequences of externally cued actions, whereas that in the ACC was highest in relation to the outcome of voluntarily chosen actions. Though the exact task performed by participants in the study by Walton et al. differs from our paradigm—and while neural activity in the OFC was localized primarily to the lateral (rather than medial) portion in their study—these previous results are broadly consistent with our finding that the integrity of the vmPFC is particularly important when stimulus values must be updated based on the observation of the consequences of externally (vs. internally) generated actions.

Though our study focused explicitly on a core component of observational learning (i.e., learning from the outcomes of externally generated actions) within a nonsocial domain, it is worth relating our findings to recent fMRI studies that have sought to reveal the neural signatures of observational learning within the social domain (Burke et al. 2010; Cooper et al. 2012; Liljeholm et al. 2012), and one study in particular that employed a closely related experimental design (Burke et al. 2010). As in phase 3 of our experiment, participants in the study by Burke et al. showed evidence of learning both from the outcomes of their own choices, but also those of another agent—in this case, a human confederate who was unknown to them. In addition, participants could also profit from the observed choices of the confederate agent (e.g., through imitation)—a source of information that was intentionally absent in our paradigm (i.e., the choices of the computer player were random), to allow us to focus squarely on learning from observed outcomes. Activity in the vmPFC in the study by Burke et al., at the time when the outcome of the confederate's choice was revealed, was found to reflect a signed prediction error signal [i.e., the difference between expected and observed outcome; also see Suzuki et al. (2012)]. Interestingly, the pattern of neural activity in the vmPFC was qualitatively different from that in the dorsolateral PFC, where activity at time of confederate choice tracked the difference between actual and expected action (i.e., an action prediction error).

By demonstrating that damage to the vmPFC impairs the ability to learn from observed rewarding outcomes, our findings are consistent with the hypothesis that the outcome prediction error signals observed by Burke et al. causally drive learning. While we characterized choice behavior using a logit model (e.g., to avoid the assumption of an exponential decay in the influence of past outcomes over time—see Materials and Methods) rather than a reinforcement learning model, our demonstration that vmPFC patients are less influenced by observational outcomes is entirely consistent with an impairment in an error-correcting learning process. Our findings, together with those of Burke et al. (2010), raise the possibility that the vmPFC forms part of the neural circuitry that supports observational learning, perhaps irrespective of whether the agent under observation is inherently social or not, a hypothesis that merits direct testing in future studies involving patients with vmPFC damage. Importantly, we believe that any vmPFC contribution to observational learning is distinct from Pavlovian learning (and the related notion of Pavlovian-instrumental transfer) where outcomes are directly experienced by the participant, and which has previously been associated with ventral striatum [e.g., see Dickinson and Balleine (2002), O'Doherty et al. (2004), Talmi et al. (2008)]—although we cannot rule out the possibility that participants imagine receiving rewards given to the computer participant, enabling a form of Pavlovian learning.

The focus of our study was the role of the vmPFC in observational learning, but we also established that all participants performed similarly when learning experientially. Notably, our finding of unimpaired experiential learning might appear surprising given the critical role ascribed to the vmPFC in value-guided decision-making (e.g., Rushworth et al. 2011; Levy and Glimcher 2012). A critical factor may be that, in our task, participants were required to choose between 2 stimuli whose magnitude varied probabilistically (cf. Daw et al. 2006) and whose reward schedules were markedly separated. As such at our probabilistic task differs in an important respect from other settings where reward magnitude is fixed at +1 or −1, and only the probability varies across stimuli (e.g., Noonan et al. 2010). Unlike these other probabilistic scenarios (e.g., reviewed in Rushworth et al. 2011), in our task it is not critical to integrate reward outcome information over long time windows. Instead, a simple strategy involving switching after a large loss would be relatively effective in our task—indeed, we observed similar rates of response switching after significant losses in all groups. This account may also account for previous findings that lesions of the vmPFC can impair experiential learning, in the Iowa Gambling Task (IGT; Bechara et al. 1994; Gläscher et al. 2012). Specifically, our paradigm and the IGT differ considerably in terms of their complexity. In the IGT participants must choose between 4 options with differing reward schedules, rendering a “lose-shift” strategy a relatively ineffective solution (though one notably employed by both vmPFC patients and comparison participants at similar rates; Bechara et al. 1994). Taken together, therefore, our findings and those from previous reports of IGT impairment after vmPFC damage suggest that the complexity of the environment (i.e., number of options and reward schedules) is a critical determinant of whether damage to the vmPFC produces an impairment in decision-making based on experiential learning. Intriguingly, this hypothesis receives support from recent work in humans which suggests that the vmPFC is only engaged when choices are sufficiently difficult (Hunt et al. 2013), and in non-human primates (Noonan et al. 2010) which demonstrates that damage to the mOFC/vmPFC only produces a significant performance deficit in a three-armed bandit task when reward schedules are closely aligned.

Along these lines, it is also worth considering whether our finding that observational learning was specifically affected by damage to the vmPFC could have arisen because it is more difficult than experiential learning (i.e., in the experiential tasks tested)—or as a result of attentional deficits in the vmPFC group. While we cannot definitely rule out such an account, we would argue that the overall pattern of findings [i.e., number of points won, RT and neuropsychological scores] does not provide support either of these accounts. First, NC and BDC participants acquired a comparable number of points over the course of experimental phases whether the task required pure experiential learning (phase 2) or pure observational learning (phase 4). Secondly, the RT of vmPFC patients was not slower in the experimental phases that involved observational learning (i.e., phases 3 and 4) compared with phases involved purely experiential learning (see Results and Supplementary Fig. 1), suggesting that all scenarios were similarly challenging. Furthermore, there was no significant group × phase interaction in terms of RT (see Results), arguing against the notion that there was an increase in task difficulty for the vmPFC group in experimental phases involving observational learning. Thirdly, the choices of the vmPFC group were influenced not only by the outcomes received but also the choices made by the computer player—refuting the possibility that vmPFC patients paid little attention during WATCH trials. Finally, neuropsychological test scores did not indicate that either group had general deficits in concentration or attention with no significant differences observed between BDC and vMPFC on relevant measures [i.e., complex figure copy, wisconsin card sorting test, trails B–A, and working memory index (WMI): all *P* > 0.1 except WMI where there was a trend for superior performance in the vmPFC group (*P* = 0.07)].

Our tasks incorporated a reversal component, and vmPFC patients performed the experiential phases of the task similarly to comparison participants in terms of overall measures (e.g., total points won and number of reversals). This contrasts with previous neuropsychological studies demonstrating a significant reversal deficit in closely related deterministic (Rolls et al. 1994; Fellows and Farah 2003) and probabilistic tasks (Hornak et al. 2004) that relied on experiential learning. For instance, a study by Fellows and Farah (2003) showed that patients classified as having primary damage to the vmPFC made more reversal errors than comparison participants in the context of a deterministic task where one response yielded a $50 win and the other a $50 loss. Notably, vmPFC patients in that study were significantly more disabled, as indexed by lower scores on a standard measure assessing IADL (mean = 17.8, SD = 3.4), than those in our cohort (mean = 20.6, SD = 0.5; nb: A maximum IADL score is 21). As such, one factor that may account for the performance difference between our cohort of vmPFC patients and those in previous reports is that the severity of damage and anatomical locus varies considerably between individual patients and groups. While these earlier reversal learning studies have typically not performed a quantitative analysis of lesion extent [Rolls et al. 1994; Fellows and Farah 2003; Hornak et al. 2004; although see Tsuchida et al. (2010)], patients characterized as having vmPFC lesions sometimes have damage extending into the lateral OFC, a region that was relatively spared in our cohort. Although it is beyond the scope of the current study, future research should directly contrast the roles of human vmPFC/mOFC and lateral OFC to determine whether these regions make unique contributions to experiential and observational learning.

One potential account of the finding of preserved experiential learning in vmPFC patients is that our study was not sufficiently powered to observe a significant impairment in experiential learning. We suggest, however, that this scenario is unlikely for 2 reasons. First, our dataset included 3000 trials per participant group (cf. 500–800 in other studies, e.g., Fellows and Farah 2003) as a result of testing a relatively large sample of vmPFC patients in a range of decision-making scenarios. Based on the magnitude of the deficit observed in previous studies [e.g., around 4000 points in the probabilistic scenario of Hornak et al. (2004)], our study would have been ideally powered to detect any impairment. Secondly, we provide “positive” evidence that patients with vmPFC damage are significantly influenced by their past rewards and choices when learning experientially.

In summary, our findings demonstrate a specific deficit in observational learning—operationalized here as the ability to learn stimulus values from the rewarding outcomes of externally generated actions—among patients with vmPFC damage. As argued above, our data provide evidence that this finding that is not easily explained by task difficulty or attentional differences between the experiential tasks used. To be clear, however, we fully concur with previous lines of work, suggesting that the vmPFC is likely critical to experiential learning in settings whose complexity or probabilistic nature (i.e., where magnitude is fixed, and probability of gain/loss varies across stimuli) differs those examined in this study [e.g., Noonan et al. 2010; Hunt et al. 2013; also see Suzuki et al. (2012)].

Interestingly, such a role for the vmPFC in updating reward representations of stimuli based on passive observation of the environment accords well with the previous work, suggesting that the vmPFC automatically computes a value (Lebreton et al. 2009) through integrating different sources of information (e.g., Smith et al. 2010; Levy and Glimcher 2012), drawing on its rich connectivity and functional interactions with sensory areas of the neocortex (Carmichael and Price 1996; Noonan et al. 2011, 2012). This contrasts with regions such as the ACC which are often believed to sustain reward representations that are more tightly coupled to specific actions mediated through more direct projections to motor areas (e.g., premotor area; Kennerley et al. 2006; Rushworth et al. 2007; Hayden and Platt 2010; Kennerley and Walton 2011; Hunt et al. 2013). While the vmPFC, therefore, appears to be necessary for observational learning, one caveat to this conclusion is that lesions that encompass this brain structure may also inadvertently damage fibers of passage which themselves might produce behavioral impairments [e.g., see Rudebeck and Murray (2011b)]. Furthermore, prior evidence suggests that other brain regions also contribute to observational learning, notably the hippocampus (Poldrack et al. 2001; Shohamy et al. 2004, 2008), a structure which is also thought to interact functionally with the vmPFC during goal-directed decision-making (Kumaran et al. 2009, 2012; Roy et al. 2012). Indeed, the “episodic” nature (i.e., extending only to *t*−1 trial) of the influence of observed rewards on current behavior accords with such a notion, and highlights the potential involvement of the vmPFC in episodic control (Lengyel and Dayan 2007). It is tempting to speculate, therefore, that the vmPFC may support observational learning through functional interactions with the hippocampus, an intriguing hypothesis that deserves investigation in future studies—perhaps drawing on recent advances in using multivariate techniques in lesion studies to identify the joint contribution of multiple brain areas to behavior (Smith et al. 2010).

## Supplementary Material

Supplementary material can be found at: http://www.cercor.oxfordjournals.org

## Funding

This work was supported by NINDS P01 NS19632 (D.T.), NIDA R01 DA022549 (D.T.), and the Wellcome Trust (Intermediate Clinical Fellowship to D.K.). Funding to pay the Open Access publication charges for this article was provided by the Wellcome Trust, UK.

## Supplementary Material

Supplementary Data
