# Supplementary material for: Damage to the Ventromedial Prefrontal Cortex Impairs Learning from Observed Outcomes
Source: Cereb Cortex. 2015 Apr 24;25(11):4504–18. doi: 10.1093/cercor/bhv080 (PMC4810001; doi:10.1093/cercor/bhv080)
Supplement: Supplementary Data [file supp_25_11_4504__index.html]

Damage to the Ventromedial Prefrontal Cortex Impairs Learning from Observed Outcomes — Damage to the Ventromedial Prefrontal Cortex Impairs Learning from Observed Outcomes — Supplementary Data 

# Damage to the Ventromedial Prefrontal Cortex Impairs Learning from Observed Outcomes

## Supplementary Data

Supplementary Data

**Files in this Data Supplement:**

- Supplementary Data - Doc file
- Supplementary Figure 1 - tif file
- Supplementary Figure 2 - tif file
- Supplementary Figure 3 - tif file
- Supplementary Figure 4 - tif file
- Supplementary Figure 5 - tif file
- Supplementary Figure 6 - tif file
