# Supplementary material for: Damage to the Ventromedial Prefrontal Cortex Impairs Learning from Observed Outcomes
Source: Cereb Cortex. 2015 Apr 24;25(11):4504–18. doi: 10.1093/cercor/bhv080 (PMC4810001; doi:10.1093/cercor/bhv080)
Supplement: Supplementary Data [file supp_bhv080_bhv080supp.doc]

**Supplemental Material**

**Title: Damage to the Ventromedial Prefrontal Cortex Impairs Learning from Observed Outcomes**

Dharshan Kumaran^1*^, David E. Warren^2*^, and Daniel Tranel^2,3^

^1^Institute of Cognitive Neuroscience, University College London, UK

^2^Department of Neurology, Carver College of Medicine, University of Iowa, Iowa City, USA

^3^Department of Psychology, University of Iowa, Iowa City, USA

*These authors contributed to this work equally.

Correspondence should be addressed to either Dharshan Kumaran (dharshankumaran@gmail.com) or David Warren (davideugenewarren@gmail.com)

**Contents:**

1. Supplemental Figure Legends (S1-S6)
2. Supplemental Tables (S1-S8)

**Figure S1** Lesion overlap maps for BDC group

Top: voxel-wise overlap maps of lesion overlap mapped into a template space. "Hotter" colors indicate greater overlap of lesions across participants (distinct color scales are used in the top and bottom panels). From left: a three-dimensional reconstruction of the lesion overlaps viewed from the right, with black vertical lines indicating the *coronal* slice locations; caudal coronal slice; middle coronal slice; rostral coronal slice.

Bottom: a schematic region-of-interest (ROI)-based lesion overlap map illustrating gray-matter lesion overlap counts in ROIs for all patients whose lesion encompassed at least 10% of ROI gray matter.

**Figure S2.** Further details of lesion overlap map of vmPFC group: Axial Slices

voxel-wise overlap maps of lesion overlap mapped into a template space. "Hotter" colors indicate greater overlap of lesions across participants. Three-dimensional reconstruction of the lesion overlaps: axial slices from ventral to dorsal (from left to right). Also see Figure 1.

**Figure S3.** Lesion map for single dPFC Case. The lesion encompassed dorsal prefrontal cortex bilaterally while excluding vmPFC. Coronal sections (top) show that the lesion included both gray and white matter, while the parcel diagrams (bottom) highlight parcels in which at 10% of the parcel’s gray matter was lesioned. See Figures 1 and S1 for corresponding lesion maps for the vmPFC and BDC groups, and Figure 1 caption for additional details.

**Figure S4.** Response Time Data

Error bars represent SEM.

**Figure S5.** Results of Logistic Regression for single dPFC Patient: Influence of previously experienced and observed rewards and choices on current behavior. See Figure 4 for details.

(**a**) vmPFC and NC groups & single dPFC patient (red dots indicate dPFC regression coefficients) (**b**) vmPFC and BDC groups & single dPFC patient (red dots indicate dPFC regression coefficients)

**Figure S6.** Overall Performance in the Deterministic task.

Bars indicate group mean performance, while points indicate individual participant performance. Unique identifying labels are provided for each patient and matched comparison pair (see Tables 1-4 for patient demographic and anatomical information).

| ID | Age | Sex | Ed. | Etiology | Chron. | Scan | IADL | IQ | WMI | WCST  Cat./PE | AVLT  5/R | CFT  C/R | TMT  B-A |
| --- | --- | --- | --- | --- | --- | --- | --- | --- | --- | --- | --- | --- | --- |
| 1 | 71 | M | 14 | R | 35 | CT | 21 | 143 | 124 | 6/4 | 14/10 | 36/32 | 37 |
| 2 | 69 | F | 16 | R | 25 | MR | 20 | 108 | 118 | 6/7 | 15/15 | 29/12^4^ | 82 |
| 3 | 48 | F | 13 | Stroke | 15 | CT | 20 | 108 | 105 | 6/10 | 10/6 | 31/15 | 17 |
| 4 | 58 | F | 16 | Stroke | 15 | CT | 21 | 115 | 108 | 6/4 | 13/6 | 36/28 | 20 |
| 5 | 57 | M | 18 | R | 14 | MR | 21 | 149 | 130^3^ | 6/9 | 15/15 | 36/31 | 30 |
| 6 | 62 | F | 14 | SAH | 12 | CT | 21 | 106 | 124 | 6/9 | 14/11 | 32/18 | 13 |
| 7 | 65 | F | 13 | R | 11 | MR | 21 | 109 | 102 | 6/7 | 15/14 | 34/24 | 21 |
| 8 | 71 | M | 12 | SAH | 12 | CT | 20 | 84 | 88 | 0/66 | 7/8 | 31/15 | 126 |
| 9 | 59 | M | 18 | R | 7 | MR | 21 | 118 | 118 | 6/11 | 14/13 | 36/28 | 35 |
| 10 | 73 | M | 16 | SAH | 4 | CT | 21 | 107^2^ | NA | NA | 8/1 | 32/9 | 29 |
| 11 | 72 | F | 12 | R | 1 | MR | 20 | 110^1^ | 127 | 6/8 | 15/12 | 33/18 | 81 |
| mean | 64.7 |  | 14.4 |  | 13.7 |  | 20.6 | 113.6 | 114.4 | 5.1/15.0 | 12.1/9.5 | 32.9/20.5 | 44.1 |
| s.d. | 7.8 |  | 1.9 |  | 9.5 |  | 0.5 | 17.2 | 13.3 | 2.0/19.0 | 3.4/4.5 | 2.3/7.7 | 36.1 |

Suppl. Table 1: Demographic and neuropsychological information for the participating vmPFC patients. Where appropriate, group means and standard deviations are presented at the bottom of columns. Abbreviations: ID, identification number; age at participation; sex; Ed., years of education; etiology (R: resection, SAH: subarachnoid hemorrhage); Chron., chronicity (i.e., time since injury) in years; scan type (CT: computerized tomography, MR: magnetic resonance imaging); IADL, instrumental activities of daily living questionnaire (Gallo et al., 2000), range 0-21; IQ, Wechsler Adult Intelligence Scale, 3rd ed. (Wechsler, 1997a) full-scale intelligence quotient except where noted; WMI, Wechsler Memory Scale, 3rd. edition (Wechsler, 1997b) working memory index except where noted; WSCT, Wisconsin Card-Sorting Task (Grant & Berg, 1998) Categories out of 6; WCST PE, perseverative errors; AVLT, Rey Adult Verbal Leaning Task (Rey, 1941) Trial 5 learning and recall scores; CFT, Rey-Osterrieth Complex Figure Task (Rey, 1941; Osterrieth, 1944), Copy and 30-minute recall scores; TMT, Trail-Making Test (Reitan & Wolfson, 1985) form A time subtracted from form B time. Notes: underlined scores are defective (patient 8 was defective on at least one measure of executive function); ^1^, these IQ scores are WAIS-IV (Wechsler, 2008) rather than WAIS-III; ^2^, WAIS-IV prorated IQ; ^3^, these patients did not have a WMS-III WMI score available, so these are WAIS-III/IV WMI scores; ^4^, this patient has a visual impairment that may have interfered with performance of the recall portion of the CFT, but has a normal memory according to other tests and reported that her impairment did not cause problems with resolving the stimuli in the current experiment.

| ID | Age | Sex | Ed. | Etiology | Chron. | Scan | IADL | IQ | WMI | WCST  Cat./PE | AVLT  5/R | CFT  C/R | TMT  B-A |
| --- | --- | --- | --- | --- | --- | --- | --- | --- | --- | --- | --- | --- | --- |
| 1 | 69 | M | 12 | S | 20 | MR | 21 | 101 | 99 | 6/9 | 10/7 | 33/21 | 39 |
| 2 | 68 | F | 20 | R | 15 | MR | 21 | 116^1^ | 115 | 6/4 | 13/8 | 31/17 | 12 |
| 3 | 47 | F | 16 | R | 14 | CT | 20 | 117 | 96 | 6/5 | 15/14 | 31/19 | 23 |
| 4 | 75 | M | 16 | S | 12 | MR | 21 | 112 | 93 | 6/8 | 13/8 | 32/13 | 36 |
| 5 | 47 | F | 16 | R | 10 | MR | 21 | 93 | 91 | 6/4 | 9/7 | 35/19 | 16 |
| 6 | 70 | F | 13 | S | 11 | MR | 21 | 95 | 85 | 3/24 | 12/7 | 21/13 | 87 |
| 7 | 61 | M | 18 | R | 10 | MR | 21 | 127 | 105 | 6/7 | 10/9 | 33/21 | 16 |
| 8 | 37 | F | 18 | R/S | 9 | MR | 21 | 118 | 108 | 6/3 | 14/12 | 36/20 | 20 |
| 9 | 67 | F | 18 | S | 8 | MR | 19.5 | 112 | 83 | 1/26 | 15/12 | 32/21 | 156 |
| 10 | 70 | M | 16 | S | 5 | MR | 21 | 117^2^ | 118 | 4/42 | 11/5 | 33/16 | 105 |
| 11 | 53 | M | 18 | SAH | 11 | MR | 21 | 123^1^ | 124 | 6/5 | 10/6 | 33/20 | 16 |
| mean | 60.4 |  | 16.5 |  | 11.4 |  | 20.8 | 111.9 | 101.5 | 5.1/12.5 | 12.0/8.6 | 31.8/18.2 | 47.8 |
| s.d. | 12.4 |  | 2.3 |  | 4.0 |  | 0.5 | 11.0 | 13.6 | 1.7/12.6 | 2.1/2.8 | 3.9/3.0 | 47.4 |

**Suppl Table 2**: Demographic and neuropsychological information for the participating BDC (brain damaged control) patients. Abbreviations: See Supplemental Table 1. **Neuropsychological information for the single DPFC case was:** age (68), sex (M), Ed (12), Etiology (R), Chron (12), Scan MR, IADL (21), IQ (117), WMI (127), WCST (3/34), AVLT (10/5), TMT B-A (18).

|  |  |  |  |
| --- | --- | --- | --- |

| ID | Hemi. | Grec | mOrbG | vmSFG | lOrbG |
| --- | --- | --- | --- | --- | --- |
| 1 | L | 0.38 | 0.11 | 0.36 | 0 |
|  | R | 0.45 | 0.50 | 0.91 | 0.44 |
| 2 | L | 0.63 | 0.27 | 0.08 | 0.07 |
|  | R | 0.60 | 0.11 | 0.26 | 0 |
| 3 | L | 0.42 | 0.31 | 0.88 | 0 |
|  | R | 0.58 | 0.49 | 0.99 | 0.33 |
| 4 | L | 0.08 | 0.06 | 0 | 0.03 |
|  | R | 0.95 | 0.93 | 0.54 | 0.45 |
| 5 | L | 0 | 0.09 | 0.55 | 0 |
|  | R | 0 | 0 | 0 | 0 |
| 6 | L | 0.52 | 0.04 | 0.46 | 0 |
|  | R | 0.33 | 0.05 | 0.34 | 0 |
| 7 | L | 0.70 | 0.52 | 0.62 | 0.15 |
|  | R | 0.73 | 0.35 | 0.94 | 0.20 |
| 8 | L | 0.63 | 0.18 | 0.73 | 0 |
|  | R | 0.01 | 0.11 | 0.55 | 0.02 |
| 9 | L | 0.83 | 0.37 | 0.83 | 0.07 |
|  | R | 0.81 | 0.17 | 0.82 | 0.02 |
| 10 | L | NA | NA | NA | NA |
|  | R | NA | NA | NA | NA |
| 11 | L | 0.74 | 0.53 | 0.15 | 0 |
|  | R | 0.81 | 0.50 | 0.42 | 0.44 |
| mean (s.d.) | L | 0.47  (0.28) | 0.22  (0.16) | 0.50  (0.31) | 0.04  (0.05) |
|  | R | 0.50  (0.33) | 0.30  (0.30) | 0.59  (0.35) | 0.16  (0.20) |

Suppl Table 3: Volumetric characterization of lesion extent in the vmPFC patients (in terms of *proportion ROI damage*). Most vmPFC patients had bilateral damage to gray matter within the vmPFC, and all had at least unilateral damage. See Suppl Table 1 for scan type and etiology. Abbreviations: Hemi., Hemisphere; vmSFG, ventromedial superior frontal gyrus; mOrbG, medial orbitofrontal gyrus; lOrbG, lateral orbitofrontal gyrus; Grec, gyrus rectus.

| ID | Hemi. | Grec | mOrbG | vmSFG | lOrbG |
| --- | --- | --- | --- | --- | --- |
| Parcel | L | 3337.00 | 8493.00 | 2704.00 | 6855.00 |
| vol. | R | 2431.00 | 8294.00 | 3138.00 | 6574.00 |
| 1 | L | 1274.06 | 975.94 | 980.16 | 0 |
|  | R | 1095.47 | 4158.28 | 2854.69 | 2919.38 |
| 2 | L | 2102.34 | 2255.62 | 208.13 | 452.81 |
|  | R | 1454.06 | 922.5 | 807.19 | 0 |
| 3 | L | 1414.69 | 2618.44 | 2383.59 | 0 |
|  | R | 1407.66 | 4085.16 | 3105.00 | 2155.78 |
| 4 | L | 281.25 | 516.09 | 0 | 220.78 |
|  | R | 2300.62 | 7685.16 | 1691.72 | 2985.47 |
| 5 | L | 0 | 756.56 | 1500.47 | 0 |
|  | R | 0 | 0 | 0 | 0 |
| 6 | L | 1743.75 | 376.88 | 1257.19 | 0 |
|  | R | 810.00 | 445.78 | 1064.53 | 0 |
| 7 | L | 2337.19 | 4412.81 | 1667.81 | 1050.47 |
|  | R | 1769.06 | 2864.53 | 2958.75 | 1341.56 |
| 8 | L | 2117.81 | 1499.06 | 1984.22 | 0 |
|  | R | 29.53 | 929.53 | 1715.62 | 133.59 |
| 9 | L | 2775.94 | 3121.88 | 2245.78 | 362.81 |
|  | R | 1971.56 | 1383.75 | 2584.69 | 244.69 |
| 10 | L | NA | NA | NA | NA |
|  | R | NA | NA | NA | NA |
| 11 | L | 2479.22 | 4502.81 | 406.41 | 492.19 |
|  | R | 1967.34 | 4151.25 | 1321.88 | 142.03 |
| mean (s.d.) | L | 1560.78  (928.25) | 1837.03  (1368.00) | 1358.59  (841.7) | 231.88  (354.44) |
|  | R | 1204.22  (807.08) | 2497.19  (2473.66) | 1864.69  (1090.96) | 1086.72  (1290.93) |

Suppl Table 4: Volumetric characterization of lesion extent in the vmPFC patients in *cubic millimeters*. Most vmPFC patients had bilateral damage to gray matter within the vmPFC, and all had at least unilateral damage. See Table 1 for scan type and etiology. Abbreviations: Hemi., Hemisphere; vmSFG, ventromedial superior frontal gyrus; mOrbG, medial orbitofrontal gyrus; lOrbG, lateral orbitofrontal gyrus; Grec, gyrus rectus.

|  | **Phase 1** | | | |  | **Phase 2** | | | |  | **Phase 3** | | | |  | **Phase 4** | | | |
| --- | --- | --- | --- | --- | --- | --- | --- | --- | --- | --- | --- | --- | --- | --- | --- | --- | --- | --- | --- |
| **Group** | **vmPFC** | **NC** | **BDC** | **dPFC** |  | **vmPFC** | **NC** | **BDC** | **dPFC** |  | **vmPFC** | **NC** | **BDC** | **dPFC** |  | **vmPFC** | **NC** | **BDC** | **dPFC** |
| **Total points scored** | 985  (222) | 968  (116) | 1116  (224) | -595 |  | 4091  (438) | 4209  (497) | 4131  (612) | -305 |  | 4505  (881) | 4439  (680) | 5226  (765) | 1156 |  | 3486  (1350) | 4127  (891) | 4528  (770) | -1020 |
| **Number of reversals** | NA | NA | NA | NA |  | 2.9  (0.4) | 3.0  (0.4) | 3.3  (0.4) | 0 |  | 2.6  (0.7) | 2.4  (0.5) | 3.4  (0.6) | 0 |  | 2.6  (0.6) | 2.5  (0.5) | 2.7  (0.6) | 0 |
| **Trials to first reversal** | *45*  *(11.3)* | *33*  *(6.2)* | *32*  *(5.2)* | 60 |  | 15.7  (2.3) | 17.2  (3.5) | 22.7  (5.9) | NA |  | 30.6  (5.8) | 34.5  (6.4) | 27.5  (5.4) | NA |  | 24.8*  (5.3) | 16.3*  (4.0) | 19.1*  (2.1) | NA |
| **Prereversal points** | NA | NA | NA | NA |  | 1260  (200) | 1435  (300) | 1337  (168) | NA |  | 1947  (332) | 2458  (370) | 1663  (227) | NA |  | 510*  (338) | 1529*  (267) | 1908*  (219) | NA |
| **Postreversal points** | NA | NA | NA | NA |  | 2830  (396) | 2774  (551) | 2794  (642) | NA |  | 2558  (992) | 1981  (758) | 3563  (852) | NA |  | 3665*  (1293) | 3339*  (916) | 2784*  (935) | NA |
| **Switching (%)** | 30.4  (4.0) | 28.0  (4.0) | 27.1  (2.9) | 32.2 |  | 26.2  (4.4) | 25.6  (3.1) | 24.8  (2.7) | 47.8 |  | 36.9  (4.7) | 37.4  (2.6) | 34.0  (3.6) | 40 |  | 33.6  (4.2) | 34.3  (3.1) | 29.9  (3.2) | 53.7 |
| **Switching after big loss (%)** | 69.8  (7.7) | 55.5  (13.3) | 66.2  (12.9) | 44.5 |  | 60.6  (7.3) | 62.5  (4.1) | 66.1  (6.3) | 40 |  | 73.5  (3.7) | 62.4  (4.1) | 74.0  (5.5) | 47.9 |  | 56.9  (3.8) | 57.8  (4.7) | 52.6  (7.0) | 50 |
| **Switching after big win (%)** | 19.4  (4.6) | 12.7  (4.5) | 10.4  (3.0) | 16.7 |  | 11.9  (3.1) | 9.9  (3.2) | 8.2  (2.6) | 41 |  | 26.2  (4.8) | 27.1  (2.8) | 23.9  (4.3) | 45 |  | 28.0  (4.8) | 27.4  (3.5) | 19.6  (3.7) | 58.2 |

**Suppl Table 5.** Summary of basic performance measures on Phases 1 (non-reversal) and 2-4 (reversal) of the probabilistic task**.** Phase 1 did not include reversal, so italicized values (Phase 1, "trials to first reversal") instead indicate trials to criterion. The criterion for reversal in Phases 2-4 was defined as choosing the good stimulus on at least 9 of the previous 10 trials. Measures of switching defined as previously**.** Prereversal points indicates the cumulative gain (i.e., total points won above the average values of both stimuli) before the first reversal trial (i.e. in the initial learning phase). Postreversal points indicates the cumulative gain (i.e., total points won above the average values of both stimuli) after the first reversal trial. No significant differences between groups (P>0.1) with the exception of the vmPFC group earning significantly fewer points in the initial learning phase (i.e., prereversal points) in Phase 4 (i.e., pure observational learning). SEMs in parentheses. *: In phase 4, several participants did not reverse at all (2 vmPFC, 2 NC, and 1 BDC & 1 dPFC). The indicated values exclude the performance of those participants.

| Descriptor | Factor | Χ^2^ | df | p |  |
| --- | --- | --- | --- | --- | --- |
| Main effect | Group | 72.02 | 50 | 0.0224 | * |
|  | Latency | 660.09 | 60 | <0.0001 | * |
|  | Participant reward | 1217.1 | 15 | <0.0001 | * |
|  | Participant choice | 1365.71 | 15 | <0.0001 | * |
|  | Computer reward | 1082.78 | 15 | <0.0001 | * |
|  | Computer choice | 97.3 | 15 | <0.0001 | * |
| 2-way Int. | Group × Latency | 61.71 | 40 | 0.0153 | * |
|  | Group × Participant reward | 9.23 | 10 | 0.5106 |  |
|  | Latency × Participant reward | 25.76 | 12 | 0.0116 | * |
|  | Group × Participant choice | 11.64 | 10 | 0.3099 |  |
|  | Latency × Participant choice | 378.6 | 12 | <0.0001 | * |
|  | Group × Computer reward | 42.34 | 10 | <0.0001 | * |
|  | Latency × Computer reward | 236.73 | 12 | <0.0001 | * |
|  | Group × Computer choice | 13.47 | 10 | 0.1985 |  |
|  | Latency × Computer choice | 41.51 | 12 | <0.0001 | * |
| 3-way Int. | Group × Latency × Participant reward | 8.16 | 8 | 0.4175 |  |
|  | Group × Latency × Participant choice | 7.5 | 8 | 0.4839 |  |
|  | Group × Latency × Computer reward | 41.05 | 8 | <0.0001 | * |
|  | Group × Latency × Computer choice | 12.43 | 8 | 0.1329 |  |
| Total | - | 3970.79 | 74 | <0.0001 | * |

**Suppl Table 6:** Logistic Regression: Results of ANOVA

The main effects and interactions of each of the following factors (i.e., predictors in the LR models) are summarized: group (discrete, levels: NC, BDC, vmPFC); latency (discrete, levels: 1-5 trials previous); participant reward (linear) and choice (discrete, A or B); computer reward and choice (see participant). * indicates significant (i.e., p<0.05) parameters. Note that the selective deficit of the vmPFC participants in observational learning is evidenced by significant Group × Computer reward, and Group × Latency × Computer reward interactions.

| **Comparison Group** | **Session** | **Phase** | **t-value** | **P-value** |
| --- | --- | --- | --- | --- |
| **BDC** | probabilistic | 1 | 2.201 | 0.052 |
|  |  | 2 | 2.093 | 0.063 |
|  |  | 3 | 1.536 | 0.155 |
|  |  | 4 | 2.079 | 0.064 |
|  |  |  |  |  |
| **NC** | probabilistic | 1 | 3.872 | *0.003* |
|  |  | 2 | 2.619 | *0.026* |
|  |  | 3 | 1.395 | 0.193 |
|  |  | 4 | 1.667 | 0.126 |
|  |  |  |  |  |
| **vmPFC** | probabilistic | 1 | 2.057 | 0.067 |
|  |  | 2 | 2.894 | *0.016* |
|  |  | 3 | 1.097 | 0.298 |
|  |  | 4 | 0.964 | 0.358 |
|  |  |  |  |  |
|  |  |  |  |  |
| **BDC** | deterministic |  | 6.045 | *0.0001* |
| **NC** | deterministic |  | 4.622 | *0.001* |
| **vmPFC** | deterministic |  | 1.398 | 0.192 |

**Suppl Table 7.** Performance of single dPFC patient compared to other patient groups. Statistics are from two-tailed modified Crawford’s t-test (df=10). Italicized P values were significant at the 0.05 level.

|  | vmPFC | NC | BDC | dPFC |  |  |  |
| --- | --- | --- | --- | --- | --- | --- | --- |
| Total points scored | 1672.7 (273.0) | 2000 (107.0) | 2081.8 (85.1) | 300 |  |  |  |
| Number of reversals | 3.8 (0.50) | 4.6 (0.24) | 4.8 (0.23) | 0 |  |  |  |
| Points before first reversal | 400 (13.5) | 436 (23.4) | 373 (20.6) | n/a |  |  |  |
| Points after first reversal | 1272 (281) | 1564 (118) | 1709 (82) | n/a |  |  |  |
| Trials to first reversal | 13 (1.6) | 11.7 (1.0) | 12.3 (0.6) | n/a |  |  |  |
| Number of errors in reversal phase | 11.9 (2.8) | 10.0 (1.1) | 9.2 (0.9) | 27 |  |  |  |

**Suppl Table 8.** Overall performance on the deterministic task. Values are group means (SEMs in parentheses). Number of errors in reversal phase refers to total number of choices of the bad stimulus after the first reversal had occurred. No significant differences were found on any measure (P>0.1: see main text). Note a single dorsal prefrontal patient was tested (dPFC).
