# Supplementary figures and images for: Damage to the Ventromedial Prefrontal Cortex Impairs Learning from Observed Outcomes
Source: Cereb Cortex. 2015 Apr 24;25(11):4504–18. doi: 10.1093/cercor/bhv080 (PMC4810001; doi:10.1093/cercor/bhv080)

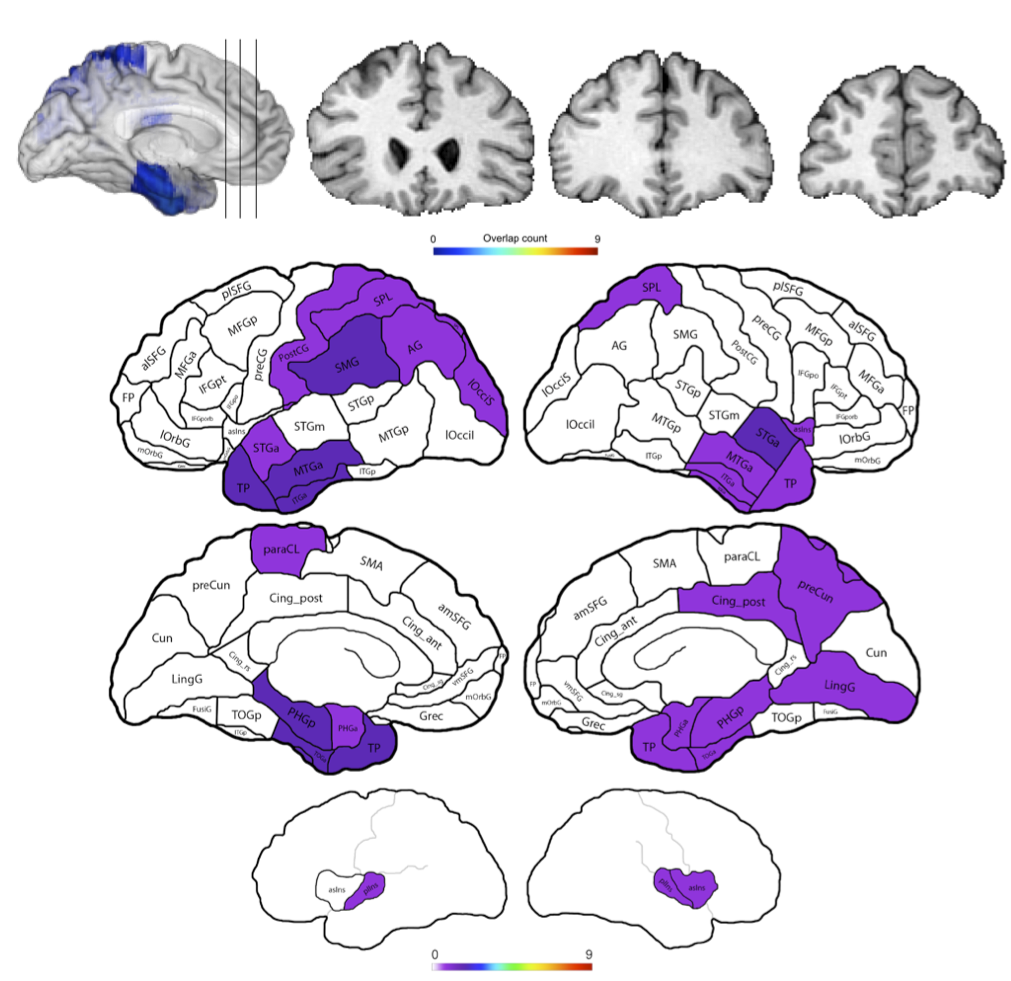

Supplement: Supplementary Data [file supp_bhv080_bhv080supp_fig1.tif]

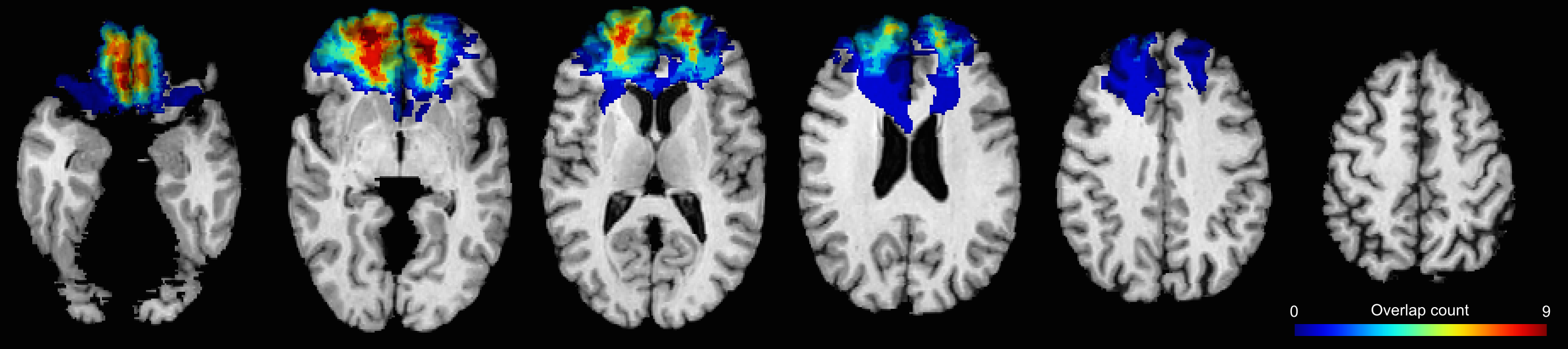

Supplement: Supplementary Data [file supp_bhv080_bhv080supp_fig2.tif]

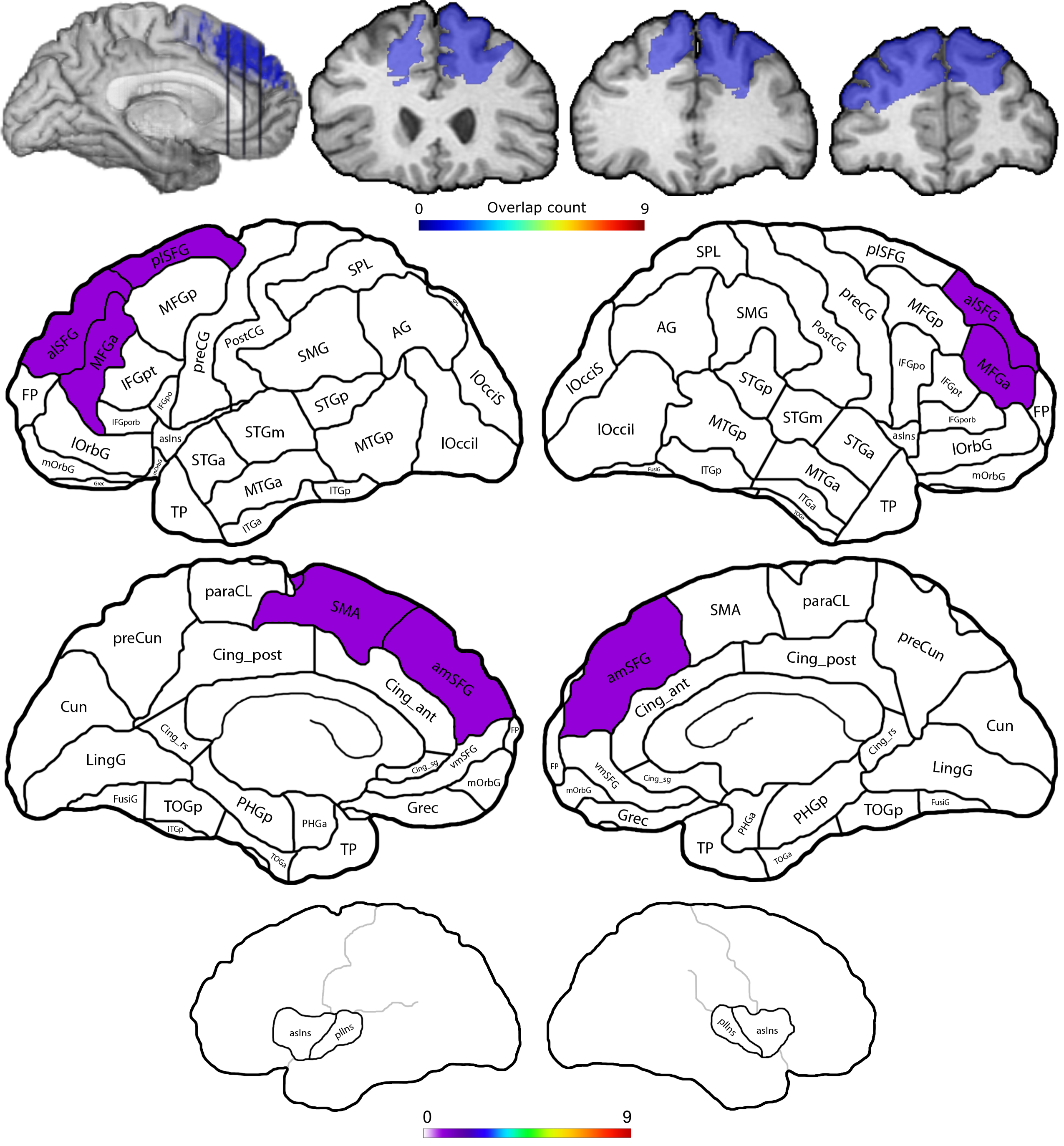

Supplement: Supplementary Data [file supp_bhv080_bhv080supp_fig3.tif]

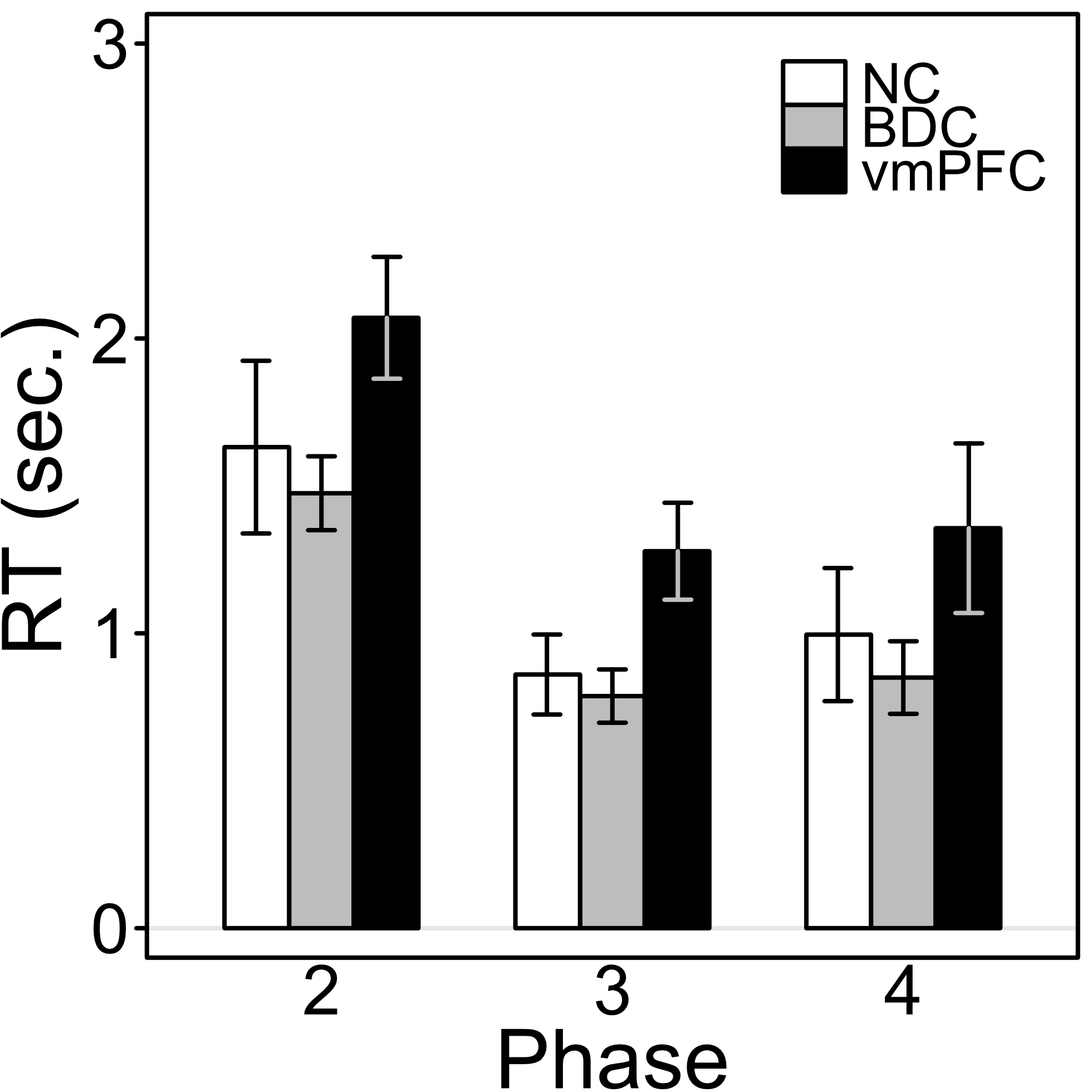

Supplement: Supplementary Data [file supp_bhv080_bhv080supp_fig4.tif]

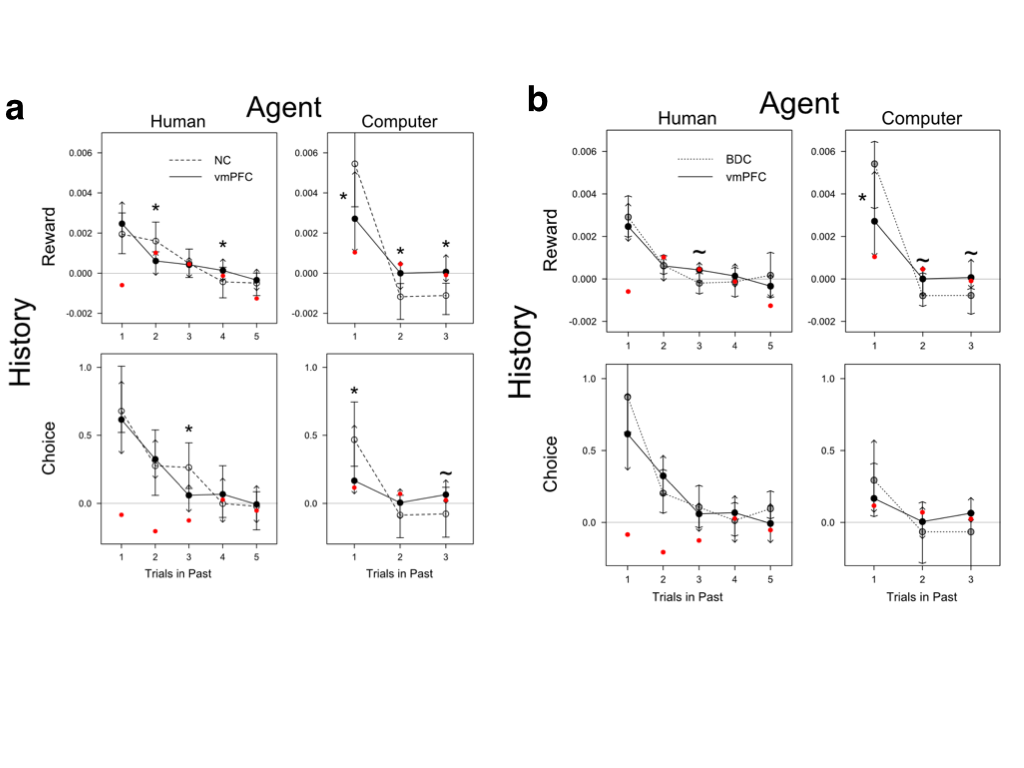

Supplement: Supplementary Data [file supp_bhv080_bhv080supp_fig5.tif]

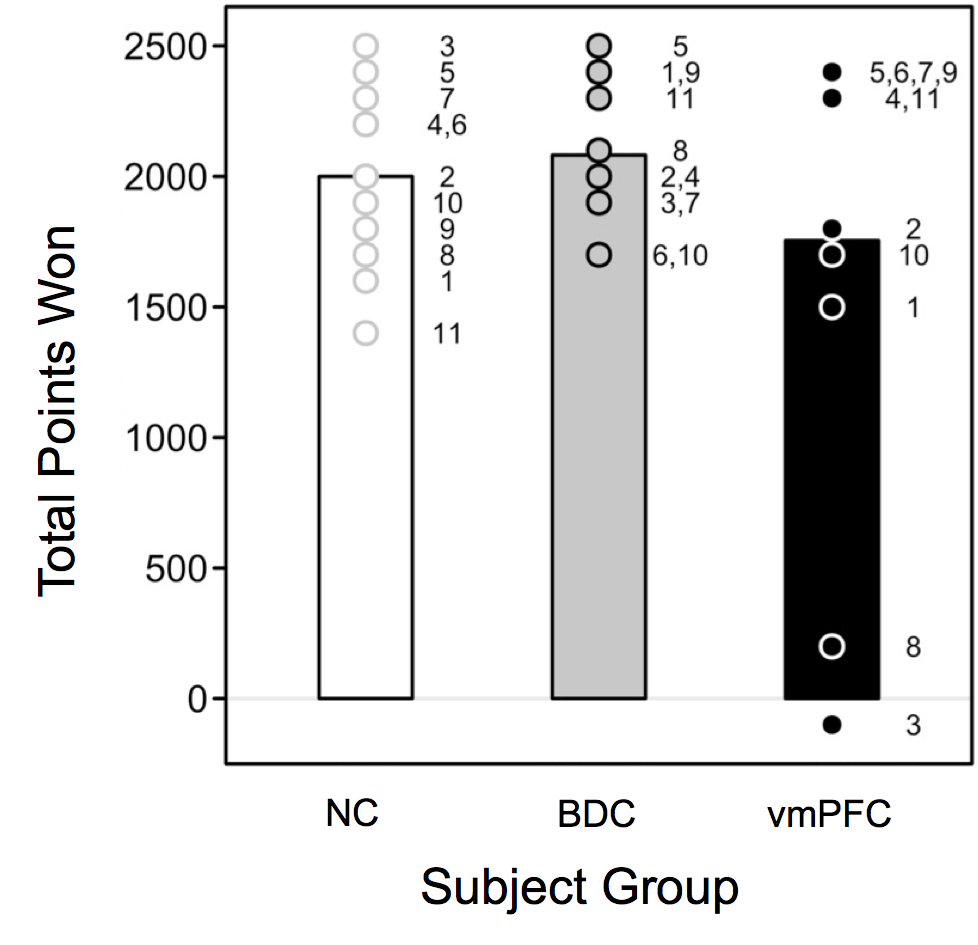

Supplement: Supplementary Data [file supp_bhv080_bhv080supp_fig6.tif]
